# Hierarchical Bayesian inference for concurrent model fitting and comparison for group studies

**DOI:** 10.1371/journal.pcbi.1007043

**Published:** 2019-06-18

**Authors:** Payam Piray, Amir Dezfouli, Tom Heskes, Michael J. Frank, Nathaniel D. Daw

**Affiliations:** 1 Princeton Neuroscience Institute, Princeton University, Princeton, New Jersey, United States of America; 2 Data61, CSIRO, Sydney, Australia; 3 Institute for Computing and Information Sciences, Radboud University, the Netherlands; 4 Department of Cognitive, Linguistics, and Psychological Sciences, Brown University, Providence, Rhode Island, United States of America; Inria, FRANCE

## Abstract

Computational modeling plays an important role in modern neuroscience research. Much previous research has relied on statistical methods, separately, to address two problems that are actually interdependent. First, given a particular computational model, Bayesian hierarchical techniques have been used to estimate individual variation in parameters over a population of subjects, leveraging their population-level distributions. Second, candidate models are themselves compared, and individual variation in the expressed model estimated, according to the fits of the models to each subject. The interdependence between these two problems arises because the relevant population for estimating parameters of a model depends on which other subjects express the model. Here, we propose a hierarchical Bayesian inference (HBI) framework for concurrent model comparison, parameter estimation and inference at the population level, combining previous approaches. We show that this framework has important advantages for both parameter estimation and model comparison theoretically and experimentally. The parameters estimated by the HBI show smaller errors compared to other methods. Model comparison by HBI is robust against outliers and is not biased towards overly simplistic models. Furthermore, the fully Bayesian approach of our theory enables researchers to make inference on group-level parameters by performing HBI t-test.

## Author summary

Computational modeling of brain and behavior plays an important role in modern neuroscience research. By deconstructing mechanisms of behavior and quantifying parameters of interest, computational modeling helps researchers to study brain-behavior mechanisms. In neuroscience studies, a dataset includes a number of samples, and often the question of interest is to characterize parameters of interest in a population: Do patients with attention-deficit hyperactive disorders exhibit lower learning rate than the general population? Do cognitive enhancers, such as Ritalin, enhance parameters influencing decision making? The success of these efforts heavily depends on statistical methods making inference about validity and robustness of estimated parameters, as well as generalizability of computational models. In this work, we present a novel method, hierarchical Bayesian inference, for concurrent model comparison, parameter estimation and inference at the population level. We show, both theoretically and experimentally, that our approach has important advantages over previous methods. The proposed method has implications for computational modeling research in group studies across many areas of psychology, neuroscience, and psychiatry.

This is a *PLOS Computational Biology* Methods paper.

## Introduction

Across different areas of neuroscience, researchers increasingly employ computational models for experimental data analysis. For example, decision neuroscientists use reinforcement learning (RL) and economic models of choice to analyze behavioral and brain imaging data in reward learning and decision-making tasks [1, 2]. The field of computational psychiatry uses these models to characterize patients and people at the risk of brain disorders [3–6]. Neuroimaging studies use models of neural interaction, such as dynamic causal modeling [7, 8], as well as abstract models to analyze brain signals [2, 9]. The success of these efforts heavily depends on statistical methods making inference about validity and robustness of estimated parameters across individuals, as well as making inference on validity and generalizability of computational models. A key theoretical and practical issue has been capturing individual variation both in a model’s parameters and additionally in which of several candidate models a subject expresses, which may also vary from subject to subject.

Computational models usually rely on free parameters, such as learning rate in RL models, which often capture quantities of scientific interest but typically vary across individuals and must be estimated from data. A dataset includes a number of subjects, and often the question of interest is to characterize parameters in a population: Is choice consistency altered in patients with attention-deficit hyperactive disorders? Do cognitive enhancers, such as Ritalin, enhance the learning rate at the population level? These questions are most naturally framed in terms of hierarchical models, which characterize both the population distributions over a model’s parameters and also each individual subject’s parameters given the population distribution. Since these two levels are mutually interrelated, they are often estimated simultaneously, using methods like expectation maximization or sampling (MCMC). For example, the hierarchical parameter estimation (HPE) procedure [10, 11] regularizes individual estimates according to group statistics, producing better individual estimates and permitting reliable group-level tests. Because subjects typically share underlying structure, hierarchical Bayesian approaches can leverage this structure to yield better individual estimates and to provide better predictions for unseen data, compared to approaches that fit each subject separately [12].

A second, and seemingly logically prior, question is which of several candidate models provides the best explanation for the data. This is important both for providing the setting within which to do parameter estimation, and also for investigating questions of scientific interest. Are rodents’ reaction times best explained by independent or competing accumulators? Do compulsive gamblers rely more on model-free RL compared to controls? Importantly, in principle (and apparently in practice) the model expressed might also vary from subject to subject; thus modern model comparison techniques rely on estimating which of several models obtains for each subject [13]. Estimating such variation is important since the prior assumption that the same model obtains across all individuals (treating model identity as a fixed effect) is a very strong (and in most cases potentially unwarranted) assumption, which makes model comparison very sensitive to outliers [13]. To estimate this variation, in turn, depends on the likelihood of each subject’s data given each model (and, thus, on each subject’s parameters for each model).

Intuitively, evaluating whether a model is a good model for a subject’s data precedes estimation of its specific parameter values; and indeed, previous research has used separate tools to solve these two problems. But statistically, the two questions are actually interconnected, because individual parameters and hence individual fit depend on which subjects belong to the population that expresses the model. Here, we address this challenge from a fully Bayesian viewpoint. This work addresses issues of statistical inference over both parameters and models, which have remained elusive with the previous hierarchical methods.

Notably, although it is accepted (for the reasons discussed above) that the best-fitting model may vary from subject to subject, hierarchical parameter estimation (conducted separately) has typically assumed that the given model is expressed over all subjects, i.e. that it is a fixed effect (and if multiple models are compared, these are each fit to the entire population). This assumption biases parameter estimation, at both individual and group levels, because it entails that the estimated parameters for each individual subject equally affect group-level estimates, even though some members of the population may be better understood as expressing altogether different models. This same bias, in turn, affects the estimation of which subjects are best fit by each model.

In this work, we introduce a hierarchical and Bayesian inference method, which solves these problems by addressing both model fitting and model comparison within the same framework using variational techniques. Furthermore, our fully Bayesian approach enables us to assess uncertainty and provide a rigorous statistical test, HBI t-test, for making inference about parameters of a model at the population level, an issue that has not been addressed in some previous hierarchical models. This paper is structured as follows. First, we highlight the main theoretical advances in our approach. A full formal treatment is given in Materials and methods and S1 Appendix. We then apply the proposed method to synthetic choice datasets as well as empirical datasets to demonstrate its advantages over previous methods.

## Results

### Theoretical results

Consider a typical computational modeling study in which data of a group of subjects have been measured and a set of candidate models are considered as possible underlying computational mechanisms generating those data. Such studies have generally two main goals: 1) to compare model evidence across competing models; 2) to estimate free parameters of models for each individual and their group-level distributions. All this is typically characterized in terms of inference in a hierarchically structured model of the data, which captures how each subject’s observations depend on their parameters and the individual parameters on their group distribution.

The HPE procedure [10, 11] employs a hierarchical approach to define the priors based on statistics of the group. This method typically assumes that for a particular model *k*, all individual parameters are normally distributed,

$$\begin{array}{c} p\left(h_{kn}\right)=N\left(h_{kn}|\mu_{k},V_{k}\right), \end{array}$$

where **h**_*kn*_ is a vector of the free parameters of the *k*th model for subject *n*, ***μ***_*k*_ and **V**_*k*_ are the mean and variance parameters, respectively, indicating the prior distribution over **h**_*kn*_.

It is important to distinguish the statistical model itself from the algorithms or approximations used to estimate it. HPE uses the expectation-maximization algorithm [14], a well-known iterative procedure, for obtaining estimating group parameters ***μ***_*k*_ and **V**_*k*_ and individual parameters **h**_*kn*_. Every iteration of this algorithm alternates two steps: 1) an expectation step in which the individual parameters are estimated in light of the group-level distribution; and 2) a maximization step in which the group parameters, ***μ***_*k*_ and **V**_*k*_, are updated given the current estimates of the individual parameters. Importantly, reflecting the assumption that all subjects express model *k*, this update weights the individual subjects’ estimates equally; for instance, the update for ***μ***_*k*_ is given by the average of subject level mean estimates (denoted ***θ***_*kn*_) across all subjects:

$$\begin{array}{c} \mu_{k}=\frac{1}{N}\sum_{n}\theta_{kn}, \end{array}$$

where *N* is the number of subjects.

Although HPE characterizes variation across subjects in the model parameters **h**_*kn*_ (that is, it treats those parameters as random effects), a critical assumption of the procedure is that the parameters for model *k* are estimated assuming that the same model is responsible for generating data in all subjects. That is, the model identity is taken as a fixed effect, in contrast to the random effects approach that assumes different models might be responsible for generating data in different subjects. The fixed effects assumption has two important implications: 1) for parameter estimation, group parameters, the group mean ***μ***_*k*_ and variance **V**_*k*_, are influenced equally by all subjects, even those who would be better fit by some other candidate model *j* ≠ *k*; 2) for model comparison, the straightforward procedure (e.g. iBIC from [10, 11]) is to compare models according to the sum of individual model evidences over all subjects, i.e. again treating the model identity as a fixed effect. Note that while it is possible to submit individual model evidence values (per subject and model) derived from HPE to a separate model comparison procedure that treats model identity as a random effect (such as random effects model selection [13]), these will be biased both from having been fit under the fixed effects assumption and also due to the optimization of the free group-level parameters. For this reason, HPE has typically been accompanied by fixed-effects model comparison [10, 11, 15], whereas attempts to study subject-subject variation in model identity [13] have typically been conducted using a different, non-hierarchical parameter estimation procedure. Altogether, violations of the fixed effects assumption can adversely influence both parameter estimation and model comparison.

Here, we extend HPE’s generative model with another level of the hierarchy, specifying for each subject which model generated their data. This is governed by a subject-specific multinomial random variable, itself drawn from a distribution controlling the proportion of each model in the population. This, in effect, merges the Bayesian model selection model from Stephan et al. [13] with HPE. To accomplish inference in this model, we then lay out a procedure for joint inference over model identities and parameters, including quantifying the probability that each model is responsible for generating data for each subject. To achieve this goal, we adopt a fully Bayesian framework in which the group parameters for each model, ***μ***_*k*_ and **V**_*k*_, are also random variables. This also gives us a straightforward way to quantify the level of certainty in group-level estimations. We use mean-field variational Bayes [16, 17], an extension of expectation-maximization [18], which is able to deal with multiple latent variables in a probabilistic model. Since HBI is a mean-field variational framework, the resulting algorithm (see Materials and methods) is an iterative algorithm. On every iteration, the HBI performs 4 steps: calculates the summary statistics, updates its estimates of the posterior over group parameters, updates its estimate of the posterior over each individual parameter and finally updates its estimates of responsibility of each model in generating each individual data. The algorithm and other important mathematical issues are given in Materials and methods. Here, we highlight three main results. The mathematical proofs are given in S1 Appendix.

As noted above, the HBI method estimates the probability of each subject’s dataset being generated by each model, or the responsibility of model *k* for generating data for subject *n*, *r*_*kn*_, which is expressed as (expected) probability. Larger values of *r*_*kn*_ (i.e. close to 1) indicate that model *k* is likely to be the true underlying model of the *n*th subject. In contrast, smaller values of *r*_*kn*_ (close to 0) indicate that model *k* is unlikely to be the underlying model for the *n*th subject. Based on the responsibilities, it is then possible to estimate the number of subjects explained by each model, ${\bar{N}}_{k}$:

$$\begin{array}{c} \;\bar{N}{\;}_{k}=\sum_{n=1}^{N}r_{kn}. \end{array}$$

Thus ${\bar{N}}_{k}$ is always less than the number of subjects and indexes the predominance of model *k* in the population. Furthermore, the fraction ${\bar{N}}_{k}/N$ is called model frequency, which always lies between 0 and 1 and is a useful and intuitive metric for model comparison.

In practice, in many situations, researchers are interested in selecting a single best model (rather than relative comparisons among several) even in the face of variation in model identity across subjects. One way to accomplish this goal is to compute the exceedance probability of each candidate model, a metric commonly used for model selection [13]. Exceedance probability is the probability that model *k* is more commonly expressed than any other model in the model space. Furthermore, the random effects approach enables us to quantify how likely the observed differences in model evidence is simply due to chance [19]. In this case, model selection is not statistically supported, as there is no meaningful difference between models. A metric called protected exceedance probability [19], which typically is more conservative than the exceedance probability, takes into account this possibility (see Materials and methods). Altogether, the random effects approach results in a more robust model comparison and model selection, one less driven by outliers than fixed-effects methods. Note that previous attempts to do model selection at group level using exceedance probability assumed no hierarchy for parameter estimation, thus did not deal with the issue that parameter estimation was not properly conditionalized by group distributions based on model identity.

We noted above that an issue with the HPE is that the influence of subjects on the group parameters is equal, due to the assumption that the model is a fixed effect. However, by virtue of its random effects structure, the comparable parameter in our approach, the mean of posterior distribution over ***μ***_*k*_, denoted by **a**_*k*_, shows an important property: Algorithmically, a subject’s effect on this parameter depends on the degree to which the model is estimated to be the underlying model for that subject. Specifically, this parameter, **a**_*k*_, is updated at each iteration as:

$$\begin{array}{c} a_{k}=\frac{1}{1+\;\bar{N}{\;}_{k}}\left(a_{0}+\sum_{n}r_{kn}\theta_{kn}\right), \end{array}$$

where ***θ***_*kn*_ is the mean of the individual posterior and **a**_0_ is the prior mean over ***μ***_*k*_. The important point in this equation is that **a**_*k*_ is a weighted average of individual parameters, in which the weights are the corresponding responsibilities, *r*_*kn*_. This is not specific to the group mean, but it is rather a general feature of our approach: contribution of model *k* to group parameters is weighted according to the responsibility of model *k* in generating data in the *n*th subject, *r*_*kn*_.

As mentioned above, another issue that has been incompletely treated in HPE is related to inference on parameters of a fitted model at the population level. Statistically, one needs the uncertainty of the estimated group mean, ***μ***_*k*_, to be able to make inference on the corresponding parameter at the group level. Since parameters fitted by the HPE are not independent but instead regularized according to the variance given by data, one cannot employ regular statistical tests, such as t-test, to test whether a specific model parameter is “significantly” different from zero. Using those tests on such parameters is biased in favor of generating a significant p-value (more false positives). The HBI framework solves this problem by quantifying the uncertainty of the posterior over the group parameter, resulting in a statistical test similar to the t-test, which we call it HBI t-test. Specifically, it is possible to show that the posterior over the *i*th group parameter in model *k*, *μ*_*ki*_, takes the form of standard Student’s t-distribution centered at the corresponding group mean, *a*_*ki*_, with $n_{k}=1+\;{\bar{N}}_{k}$ as degrees of freedom. The resulting t-value takes an intuitive form:

$$\begin{array}{c} t=\frac{\mu_{ki}-a_{ki}}{s_{ki}/\sqrt{n_{k}}}, \end{array}$$

where *s*_*ki*_ is the empirical deviance statistics for the *i*th parameter of model *k*. Therefore, $s_{ki}/\sqrt{n_{k}}$ plays the role of standard error, which we call it hierarchical error. Note that the degrees of freedom of the test depend on the number of subjects (i.e. evidence) in favor of model *k* given by ${\bar{N}}_{k}$, not the total number of subjects. Other group statistics, *a*_*ki*_ and *s*_*ki*_, are also weighted according to the responsibilities of model *k* in generating data of each subject (as formally obtained in Materials and methods). Using this marginal distribution for population-level group parameters, the HBI t-test enables researchers to determine whether a parameter is significantly different from an arbitrary value, notably 0. For example, the parameter is significantly different from 0 at *P* < 0.05 if 0 does not fall within the 95% credible interval.

### HBI for model comparison and parameter estimation

In this section, we apply the proposed HBI method to synthetic datasets and compare its performance with that of HPE, as well as with a non-hierarchical inference (NHI) method estimating parameters for each subject independently according to some fixed, a priori Gaussian priors [20–23]. Importantly, these methods differ in their statistical assumptions about the generative process of data. The NHI assumes no hierarchy in parameter estimation. We then used the individual-level evidence approximated by the NHI (S1 Text) to subsequently perform random effects model comparison using the procedure introduced by Stephan et al. [13, 19]. This means that whereas the NHI procedure assumes no hierarchy across parameters, it does (via the Stephan procedure [13]) allow for a hierarchical structure over model identity. In contrast, the HPE procedure, as introduced by Huys et al. [10, 11], assumes a hierarchy over parameters, but no hierarchy over model identity: we accordingly, use it with a fixed-effects model comparison procedure. The HBI assumes that both parameters and model identities are generated hierarchically in turn. Note that related approximations, as similar as possible, have been used for making inference in these methods, which allows for a fair comparison (S1 Text) since our main points concern the statistical structure of the methods, not the estimation techniques. In particular, HPE builds upon NHI’s Bayesian inference of per-subject parameters to condition these on additional group level parameters, by using expectation-maximization [14]; and HBI extends that algorithm to condition these on an additional level of model identity variables, by using variational Bayes [16, 17]. We also use the same (Laplace) approximation to marginalize the subject-level variables in all three methods. The HBI algorithm has been given in Materials and methods and details of implementing the NHI and HPE have been given in S1 Text. The details of simulation analyses and parameters used in simulations have also been given in S1 Text.

The HBI is general and could be applied to any type of data, such as choice data, reaction times, physiological signals and neural data. Since we are primarily interested in models of choice data, we focus on decision-making experiments.

#### Model comparison and parameter estimation for models with the same number of parameters

First, we considered a relatively easy problem in which the number of parameters in models is the same. We simulated a dataset including 40 artificial datasets using two different learning models and a randomly generated reward sequence (binarized Gaussian random-walk). Both models maintain a value for each of the two possible actions and calculate a prediction error signal representing the difference between the seen reward and predicted value. On every trial, the action value gets updated according to the product of the prediction error and a learning rate. The first model is an RL model, in which the learning rate is a constant free parameter, *α*. The second model is a Kalman filter model in which the learning rate gradually decreases on every trial. The decreasing rate depends on a positive free parameter (representing observational noise), *ω*. Both models employ a softmax function together with an inverse-temperature parameter, *β*, to calculate the probability of each action according to corresponding expected values. Therefore, both models contain two free parameters and neither of them is nested within the other one. The RL and Kalman filter models were then used to simulate 10 and 30 artificial datasets, respectively. Parameters of these models were drawn randomly from normal distributions. Since parameters of these models have theoretical constraints, we used appropriate functions (sigmoid or exponential) to transform these randomly generated parameters. Using this procedure, we constructed a dataset of 40 artificial subjects, in which the true underlying model is known. We applied the HBI to this dataset to estimate parameters and model evidence given the sequence of actions. Simulations were repeated 20 times.

**Fig 1:**
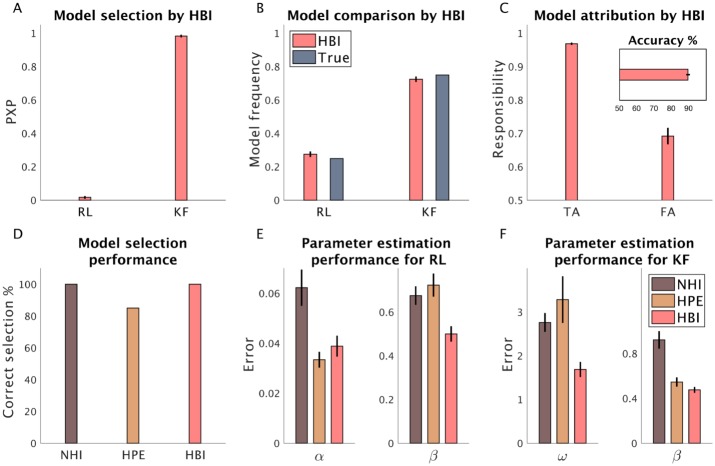
Performance of the HBI in a synthetic dataset. 10 and 30 artificial subjects were generated according to the RL (RL) and Kalman filter (KF) models, respectively. A) Model selection by HBI using protected exceedance probabilities (PXP); B) Model frequencies estimated by the HBI. C) Model attribution at the individual level by the HBI; Responsibility estimates are plotted for true attributions (TA), in which the true model has been attributed, and for false attributions (FA), in which the incorrect model is attributed. The HBI shows lower levels of responsibility for FA. Inset: percentage of correct assignment of the model by the HBI at the individual level. D) Comparison of accuracy of model selection with HPE and NHI; E, F) Error in estimating individual parameters of the RL (E) and the Kalman filter model (F). The estimation error is defined as the absolute difference between estimated parameters and the true parameters. In all plots, error-bars are standard errors of the mean obtained across 20 simulations.

Fig 1 shows the results of applying the HBI on this dataset. We first reported protected exceedance probability (Fig 1A), a metric commonly used for Bayesian model selection [19], which is the probability that each model is the most likely model across all subjects taking into account the null possibility that differences in model evidence are due to chance. This analysis revealed that the HBI has correctly identified the Kalman filter as the most likely model across the artificial datasets in all simulations with probability close to 1. Next, we looked into model frequency, which represents the ratio of subjects assigned to each model. As plotted in Fig 1B, model frequencies estimated by the HBI is close to true frequencies, 0.25 and 0.75 for the RL and Kalman filter models, respectively (Fig 1B). We then examined the HBI performance in model attribution at the individual level (Fig 1C). The HBI attributes models to each individual by quantifying responsibility parameters, which is the probability that that model is the true underlying model for that individual. First, we verified that the HBI has assigned the correct model to about 90% of all subjects (Fig 1C, inset). We then looked into the average of responsibilities for true attribution (those cases whose model was correctly identified) and for false attribution (those cases whose model was erroneously assigned) (Fig 1C). We found that the average of responsibilities estimated by HBI is about one for true attributions and it is closer to chance-level (than one) for false attributions. This means that the HBI method was quite certain when it was successful in identifying the true model and uncertain in cases in which it failed to recognize the true model. Later, we will examine HBI performance in model attribution more thoroughly.

We then compared the performance of the HBI with the HPE and NHI. Note that NHI depends on Gaussian priors over parameters. Across all simulations and models, we used the same Gaussian prior (with mean 0, and variance 6.25, similar to our previous works [24]). This value for the prior variance ensures that parameters can vary in a wide range with no substantial effects of prior (see S1 Text for a formal derivation). The hierarchical methods, in contrast, replace NHI’s fixed prior over individual-level parameters with additional group-level parameters that are themselves estimated from the data.

In this set of simulations, all methods performed well in recognizing the most likely model (i.e. the Kalman filter) across all samples (Fig 1D) at the liberal threshold of 50%, although the HPE performed worse than the other two models (failing 15% of simulations). In the next section, we examine the limitations of HPE for model comparison more thoroughly.

We then investigated the performance of these methods in parameter estimation. We quantified individual-level estimation error, which is defined as the absolute difference between estimated individual-level parameters using that method and true individual-level parameters used for generating data. For both models and all parameters, the average error in parameter estimation by HBI was smaller than those by HPE and NHI (Fig 1E and 1F). Furthermore, HPE performed better than NHI in estimation across all parameters. These results were indeed theoretically expected. Unlike NHI, both HPE and HBI use group statistics to regularize parameter estimation for each individual. However, while HPE uses all subjects equally to regularize group parameters of a model, HBI weights individuals according to its responsibility (i.e. its belief that that model is responsible for generating each individual dataset).

#### Robustness of model comparison to outliers

We noted before that fixed effects model comparison using HPE is very sensitive to outliers. This is because fixed effects approaches sum up evidence across all subjects. If a few outlier subjects show large evidence in favor of a model, those usually impact model comparison adversely. In contrast, the HBI takes a random-effects approach, in which the contribution of every subject in favor of each model is normalized according to the corresponding responsibility, which is a relative evidence measure with a maximum of one. In this section, we show a simulation analysis to demonstrate this point.

We took the same datasets generated in the previous simulations by the RL and Kalman filter models. We then identified one outlier subject in that dataset that showed the largest evidence in favor of the RL model. From all 200 subjects generated using the RL model across all 20 simulations in the previous analysis, the subject with maximum relative log-likelihood in favor of the RL model (under the HPE parameters) was selected as the outlier subject in evidence space (the relative log-likelihood for this subject was 4 times more than average relative log-likelihood). This outlier subject was then used to create datasets with 1, 2 or 3 outliers by copying it 1, 2 or 3 times, respectively, and adding those copies to the original dataset.

**Fig 2:**
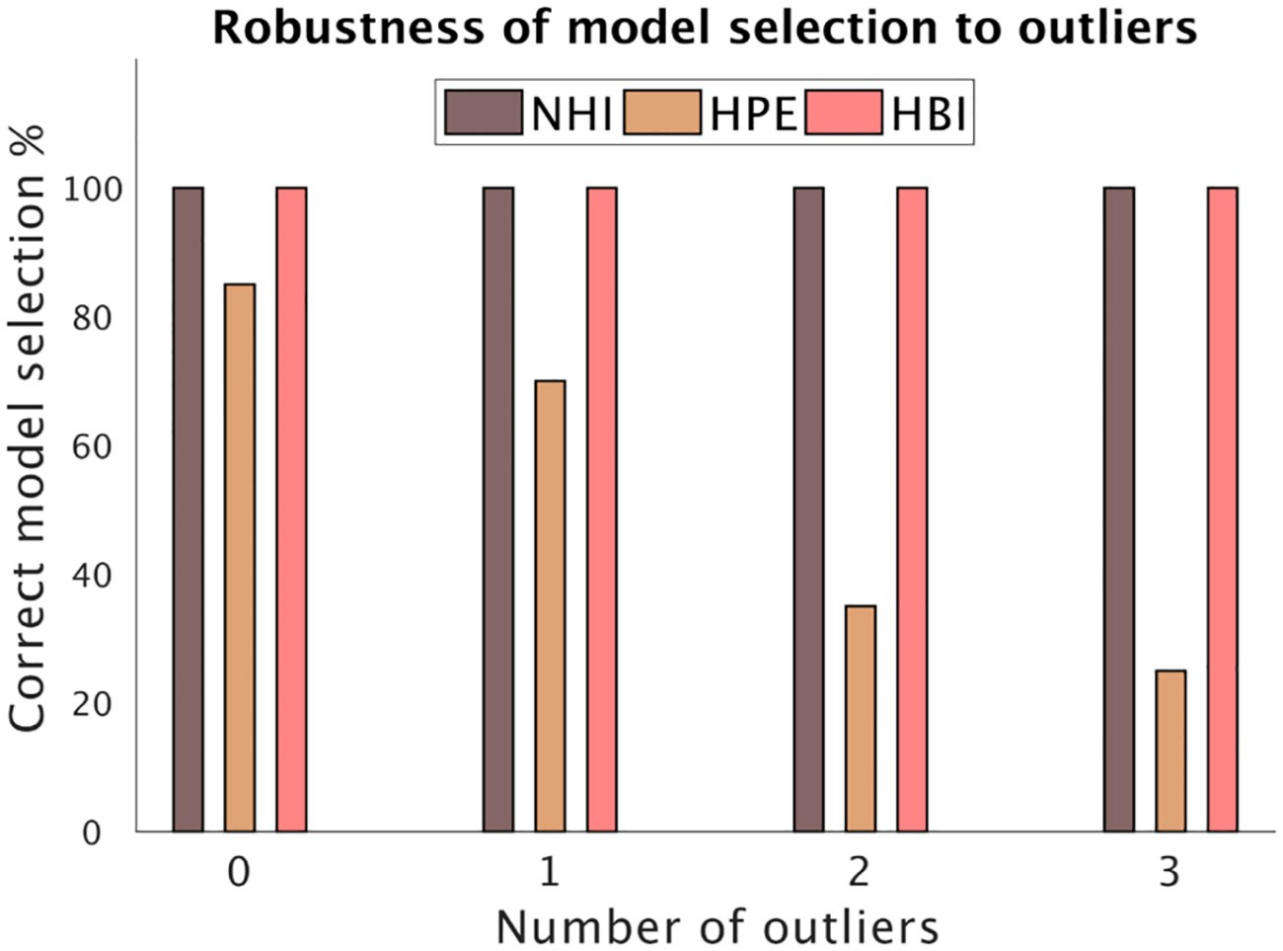
Robustness of model selection to outliers. The same 20 datasets simulated in the previous section were used as the base datasets (i.e. 0 outliers) and the effects of adding 1, 2 or 3 outliers to each dataset were examined. The HPE shows severe sensitivity to outliers, while the other two (random effects) methods are robust.

We then compared the performance of NHI, HPE, and HBI. Note that while NHI and HBI perform random effects model comparison, HPE performs a fixed effects model comparison. As shown in Fig 2, whereas the performance of HPE is very sensitive to outliers, the random effects model comparison of NHI and HBI are robust. Note that although NHI performs well in the model selection here, we will demonstrate its limitations for model comparison in the next section. It is also important to note that the outlier here is in the space of model evidence (i.e., a subject displaying abnormally large evidence for one model over another). We will examine the effects of outliers in parameter space later.

#### Model comparison and parameter estimation in models with different number of parameters

We then considered a challenging problem in which the number of free parameters in two models is different and one model is a special case of the other one. Such problems are ubiquitous in studies using computational models and inference using hierarchical approaches is typically even more advantageous in this setting, as the variance explained by such models are more likely to overlap.

The first model was again assumed to be an RL model with a constant learning rate parameter, *α*. The second model, however, was assumed to contain two different learning rates depending on whether the prediction error is positive or negative (dual-*α* RL), commonly used to assess asymmetries in learning from positive vs negative prediction errors [25, 26]. Both models use the same choice function, i.e., a softmax function with an inverse-temperature parameter, *β*. The RL and the dual-*α* RL models were then used to simulate 10 and 30 artificial datasets, respectively. Note that the RL model is a nested case of the dual-*α* RL, in which *α*^+^ = *α*^−^.

**Fig 3:**
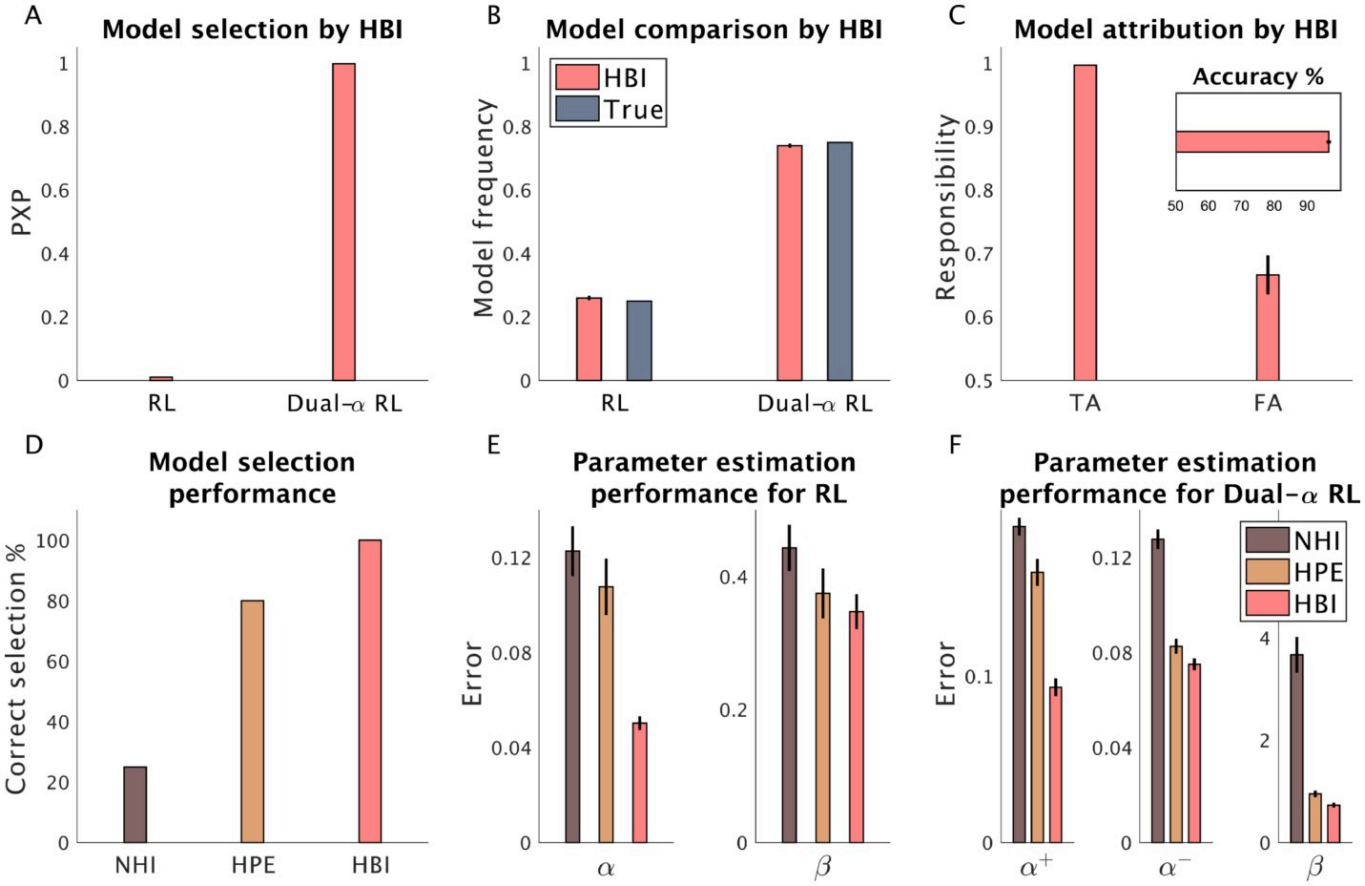
Performance of the HBI in a synthetic dataset including models with the different number of parameters. 10 and 30 artificial subjects were generated according to the RL and dual-*α* RL models, respectively. A) Model selection by HBI using protected exceedance probabilities (PXP); B) Model frequencies estimated by the HBI. C) Model attribution at the individual level by the HBI. Responsibility estimates are plotted for true attributions (TA) and for false attributions (FA). The HBI shows lower levels of responsibility for FA. Inset: percentage of correct assignment of the model by the HBI at the individual level. D) Model selection performance of NHI, HPE, and HBI; E, F) Error in estimating individual parameters of the RL (E) and the dual-*α* RL model (F). The estimation error is defined as the absolute difference between estimated parameters and the true parameters. In all plots, error-bars are standard errors of the mean obtained across 20 simulations.

As Fig 3 shows, the HBI method was successful in model selection (i.e. recognizing the most likely model, Fig 3A). Model frequencies estimated by the HBI are close to true frequencies, 0.25 and 0.75 for the RL and dual-*α* RL models, respectively (Fig 3B). At the individual level, HBI assigned the correct model to each individual in 95% of all subjects and was also quite certain when it was successful in selecting the right model (Fig 3C). In contrast, in those rare cases in which HBI failed to recognize the correct underlying model (false attributions), it assigned responsibility that was only slightly above chance.

Next, we compared the performance of the HBI with that of NHI and HPE. Here, NHI fails to choose correctly the most likely model in 75% of simulations (Fig 3D). This is likely because non-hierarchical methods penalize more complex models more harshly than do their hierarchical counterparts because they neglect the structure of the data. In particular, the issue is that a model with one additional parameter adds one independent free parameter per subject in the non-hierarchical case, which carries an excessive overfitting penalty, whereas these parameters are pooled by being drawn from a common distribution in the hierarchical setting, ensuring less overfitting and a more moderate complexity penalty. Note that reducing the variance of the prior of the NHI decreases the complexity penalty and somewhat improves model selection performance slightly in this scenario, but it also worsens parameter estimation (S1 Fig). This poor parameter estimation has negative consequences also for model selection in other situations in which the RL should be favored (S1 Fig). Therefore, in general, the NHI is not flexible enough to capture the true model in different situations.

We can also consider why the estimation errors of HBI are much smaller than those of HPE. Consider, for example, the learning rate parameter of the RL model, *α* (Fig 3E). In generating the datasets for this analysis, *α* was assumed to be smaller than the learning rate parameters of the dual-*α* RL model. This structure was designed to exercise a situation in which the HBI excels, and the HPE has trouble: when the parameters systematically differ across models, and therefore failing to take into account which subjects exemplify which model confuses the parameter estimates. In particular, since the HPE uses average statistics across all subjects (even those generated by the dual-*α* model) to constrain parameters, the group average estimate of *α* by HPE was much larger than the true average. Therefore, the individual estimates of *α* by HPE are also tended to be larger than the true parameters, resulting in larger estimation error. The HBI does not have this problem because the group statistics are estimated using a weighted average, in which the weights are the corresponding responsibilities of models. Note that for a different set of learning rate parameters, in which the learning rate of the RL is in the middle of those of dual-*α* RL model, and the consequences of estimating parameters across all subjects thus less problematic, the difference between the HPE and HBI might not be so pronounced (S2 Fig).

**Fig 4:**
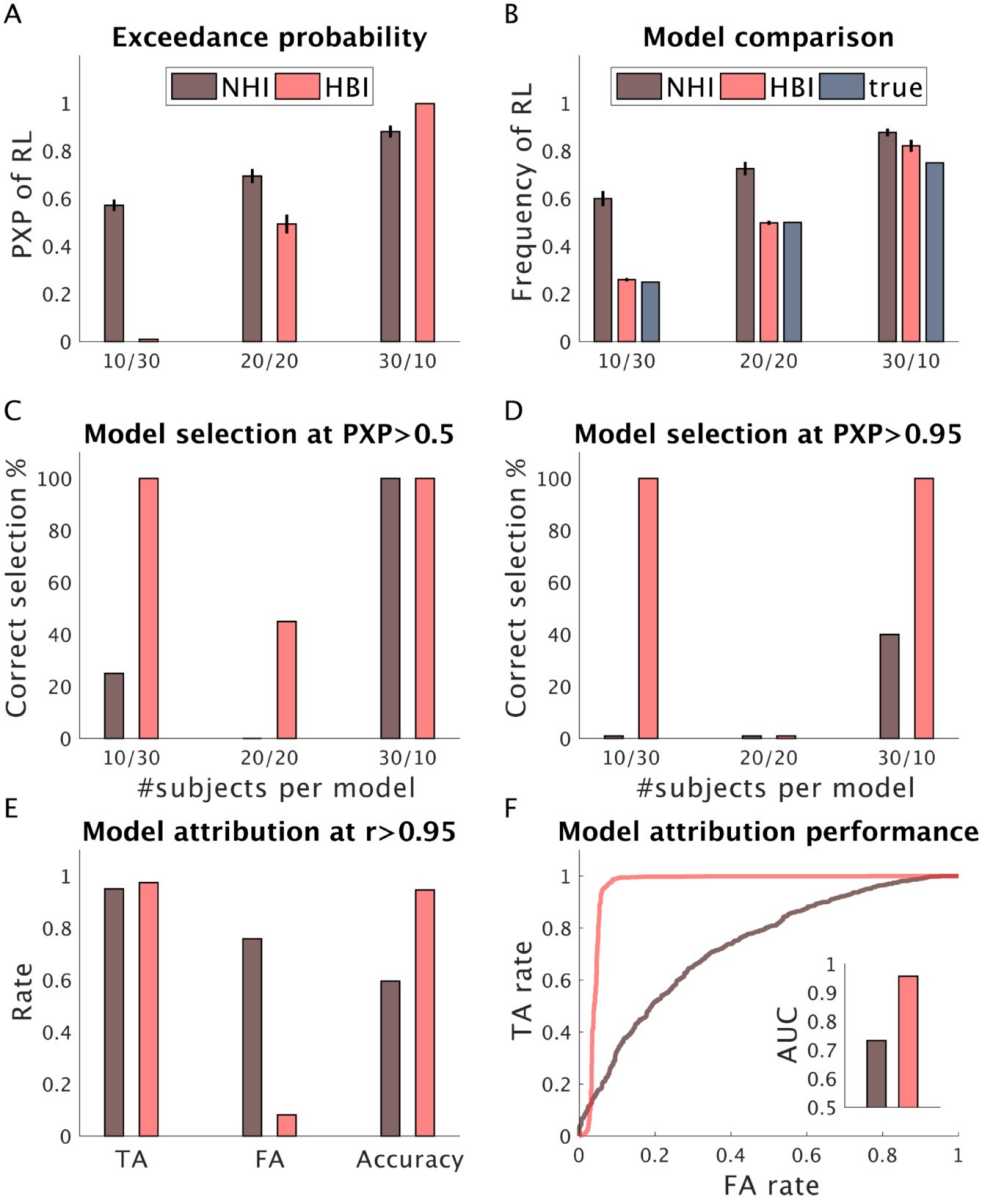
Comparison of HBI with NHI in model selection and model attribution. We compared the performance of HBI and NHI in three simulation analyses with different ratio of subjects expressing each model. The first simulation includes 10 subjects expressing RL and 30 subjects expressing dual-*α* RL model (10/30). The second one includes 20 subjects per model (20/20) and the third one includes 30 subjects expressing RL and 10 dual-*α* RL (30/10). A) Mean protected exceedance probabilities (PXP) estimated by the HBI and NHI; B) Mean model frequency of RL across all simulations (true frequencies are also plotted). C-D) Model selection performance at PXP>0.5 (C) and PXP>0.95 (D). For the 20/20 simulations, 50% of each model should be selected at the chance level, i.e. PXP>0.5, and none of the models should be selected at PXP>0.95. E) Model attribution performance, at the individual level, using responsibility (*r*) parameters at 0.95 thresholds across all three simulations. The HBI is more accurate than the NHI in model attribution and shows more true attributions (TA) and less false attributions (FA). E) ROC curves, across all three simulations, for HBI and NHI, which illustrate model attribution performance at various threshold settings. Inset: area under the curve (AUC) of the ROC, as a metric for model attribution performance. The HBI shows better performance than the NHI according to this metric. In A-B, error-bars are standard errors of the mean obtained across 20 simulations.

So far, we conducted model selection using a liberal threshold (50%). Often researchers are interested to perform model selection using higher thresholds of exceedance probabilities. With higher thresholds, we expect that none of the models get selected in situations in which there are equal numbers of subjects expressing each model. As both HBI and NHI (but not HPE) compute exceedance probabilities and model frequencies, we compared their performance in model selection. Here, we considered different ratios of subjects expressing each model. In particular, in addition to the previous simulation in which the RL model was less frequent, we considered two other situations in which the ratio of subjects expressing each model was equal or was more in favor of the RL model (Fig 4). These analyses showed that HBI is superior to the NHI, as its protected exceedance probabilities are closer to one when one of the models is actually more frequent. The HBI model frequency is closer to the true frequencies than the NHI. Furthermore, the HBI selects the most likely model with higher exceedance probabilities. It is important to note that NHI overestimates model frequencies in favor of the RL model in all simulations, probably again due to additional overfitting (and correspondingly higher penalties for the additional parameter) in the non-hierarchical setting.

We then examined the performance of HBI and NHI in model attribution at the individual level (Fig 4E). The HBI computes responsibility parameters for every subject and model, which is the posterior probability that that model generated the data for that subject. Similar parameters can be estimated using evidence approximated by the NHI. Using the threshold of 0.95 for responsibilities (*r* >0.95), we observed that the HBI is more accurate than the NHI in model attribution. This is mainly because the NHI shows a higher false attribution rate due to its bias to attribute individuals to the simpler model. Note that it is possible to compute true attribution and false attribution rate using different thresholds for responsibilities here. In machine learning, it is common to illustrate attribution performance of a binary classification machine using plots called receiver operating characteristic (ROC) curves, which are obtained by plotting the true attribution rate against the false attribution rate at various thresholds. In ROC curves, the upper left corner point (i.e. 0 false attribution rate, 1 true attribution rate) represents perfect classification. The diagonal line, on the other hand, represents classification at the chance level. The area under the curve in this plot is, therefore, a good metric for classification performance. This metric shows that the overall model attribution performance of the HBI is better than that of NHI (Fig 4F).

#### Effects of number of trials

**Fig 5:**
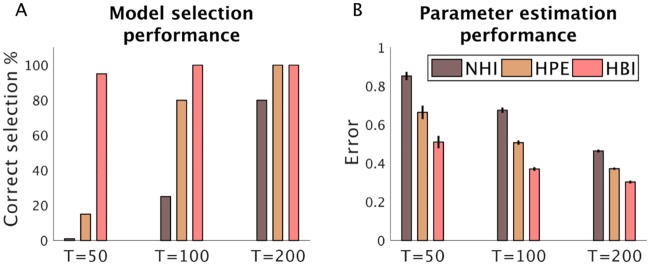
Performance of the HBI as a function of the number of trials. 10 and 30 artificial subjects were generated according to the RL and dual-*α* RL models, respectively. These simulations were performed with a different number of trials (T) per subject. A) The accuracy of model selection by NHI, HPE, and HBI for T = 50, T = 100, and T = 200 trials; B) Mean error in estimating individual parameters across both models and parameters. Note that the estimation errors here are computed on the normally distributed parameters. The estimation error is defined as the absolute difference between estimated parameters and the true parameters. In all plots, error-bars are standard errors of the mean obtained across simulations 20 times.

It is also important to note that all these methods are sensitive to the amount of within-subject data (i.e. the number of trials). Importantly, HBI is even more useful when there are a limited number of trials (Fig 5). In this case, non-hierarchical methods, such as NHI, over-penalize complex models even more, as there are fewer data-points per subject to justify additional parameters. Furthermore, in this case, the HPE model selection performance is even more sensitive to outliers, as outliers are more likely when data per subject is limited. Therefore, the HBI performs better than the other two methods in model selection when there is limited within-subject power (Fig 5A). Hierarchical methods are also more powerful in parameter estimation in this case, although the HBI performs better than the HPE across a different number of trials (Fig 5B).

#### Effects of number of participants

**Fig 6:**
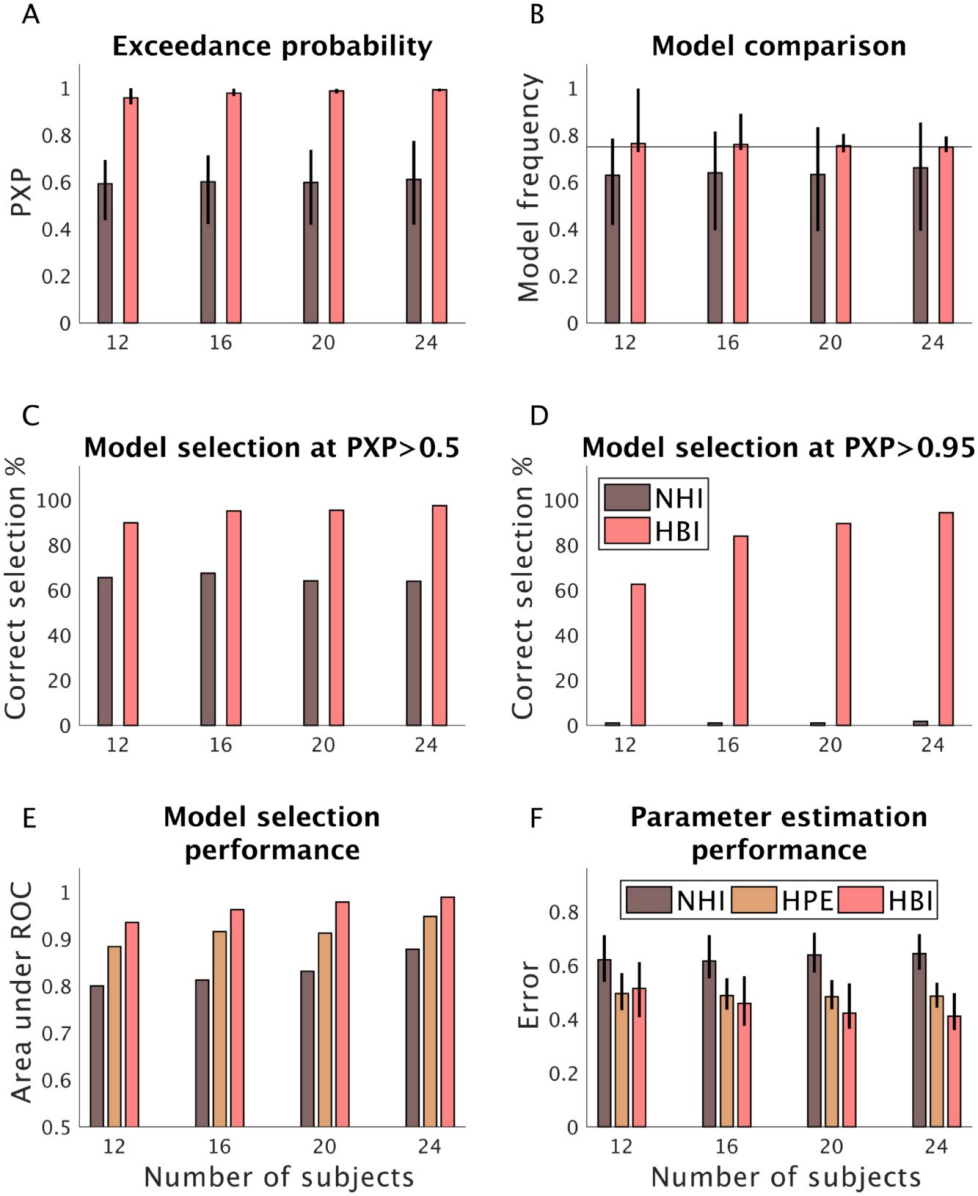
Performance of the HBI as a function of the number of subjects. In this analysis, simulations were repeated 1000 times, in which in half of the simulations, the ratio of the RL model was three times more than the dual-*α* RL, and vice versa in the other half. A) Protected exceedance probabilities (PXP) of the most frequent model estimated by the HBI and NHI; B) Model frequency of the most frequent model across all simulations. The black line indicates the true frequency (0.75). C-D) Model selection performance by the HBI and NHI at PXP>0.5 and PXP>0.95, respectively. The NHI almost never selects the most frequent model at PXP>0.95. E) Model selection performance using area under the ROC curve. Higher values indicate better performance (one corresponds to perfect model selection). The HBI performance improves by increasing the number of subjects. F) Error in estimating individual parameters across both models and parameters. Estimation errors are computed on the normally distributed parameters. The estimation error is defined as the absolute difference between estimated parameters and the true parameters. In A, B, and F, median across 1000 simulations is plotted and error-bars represent the first and third quantile.

Hierarchical methods are also sensitive to the amount of between-subject data (i.e. the number of subjects expressing each model). Moreover, model selection can be particularly unstable with a small number of subjects. Therefore, we did another simulation analysis with a smaller number of subjects and tested the performance of HBI in model selection. We performed a simulation analysis with the RL and dual-*α* RL models, in which we manipulated the number of subjects. We repeated simulations 1000 times, in which in half of the simulations, the ratio of RL model was three times more likely than the dual-*α* RL, and vice versa in the other half (Fig 6). These simulation analyses showed that the HBI selects the more frequent model with a high protected exceedance probability. The model selection performance of the HBI improved with a higher number of subjects. Across all simulations, the NHI estimates protected exceedance probabilities that are only slightly above chance and it fails to select the more frequent model.

Next, we compared model selection performance of all three methods using the area under the ROC curves for a different number of subjects (Fig 6E). Here, model selection of NHI and HBI was performed using protected exceedance probabilities. For HPE, the normalized evidence (i.e. normalized Bayes factor) was used for model selection. The HBI performed better than the other two methods with a higher area under the curve. Finally, we compared the parameter estimation performance of these methods (Fig 6F). Across all parameters and subjects, the average estimation error in individual-level parameters was quantified. The analyses showed that the HBI exhibits lower estimation error than the other methods and its performance improves when there is a higher number of subjects.

#### Robustness of parameter estimation to outliers

All model fitting methods are sensitive to outliers whose parameters are dramatically different from other subjects. Although HBI is more robust than HPE against outliers in evidence space, there is no theoretical reason that HBI is more robust against outliers in parameter space. Indeed, both HPE and HBI make the distributional assumption that subjects’ parameters vary according to a Gaussian distribution, and outliers (or indeed other non-Gaussian structures) violate this assumption. However, since the HBI takes into account multiple models during fitting, it is possible to reduce the effects of outliers on estimated group parameters in another way, by including additional simple models in the model space to “soak up” these subjects. Defining such a simple model depends on the nature of data and task. For example, in learning tasks, outliers typically show no learning effect (resulting in a decision noise parameter of about zero) or simple strategies such as switching decisions according to the most recent outcome (value is always equal to the most recent outcome). A simple model that captures both those situations is a softmax that translates the most recent outcome to probabilities according to a decision noise parameter. If the decision noise parameter is zero, this model captures outliers that outcomes have no effect on their choices.

**Fig 7:**
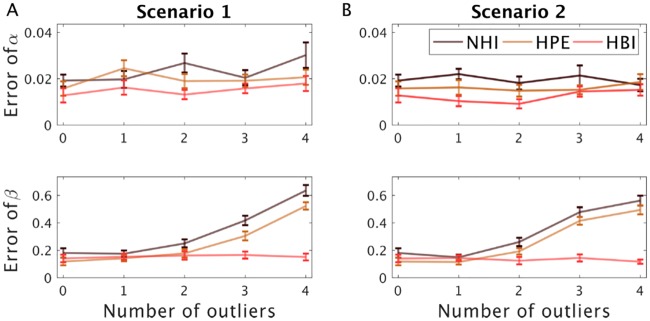
The sensitivity of parameter estimation to outliers. 30 subjects are simulated using the RL model. A) In scenario 1, a number of outliers are also simulated with the same learning rate but small decision noise parameter. B) In scenario 2, outliers are simulated with small learning rate and small decision noise parameter. Errors in recovering the group-level parameters are plotted (for the learning rate, and decision noise,). HBI performs better than alternatives. The estimation error is defined as the absolute difference between estimated group-level parameters and the true parameters. In all plots, error-bars are standard errors of the mean obtained across simulations 20 times.

We considered two scenarios to demonstrate this point experimentally (Fig 7). In the first scenario, 30 subjects were generated according to the RL model and a number of outliers that were generated by using the same model with the same learning rate but a small decision noise. We then used the HBI with a model space including an RL model and the simple model described above. We found that the estimation error for capturing the group mean was smaller for the HBI than the NHI and HPE methods. In the second scenario, we considered a more realistic situation in which outliers were generated based on a small learning rate and a small decision noise. Similar to the previous simulation, HBI exhibited less estimation error for group parameters compared with other methods.

#### HBI for model spaces with more than two models

**Fig 8:**
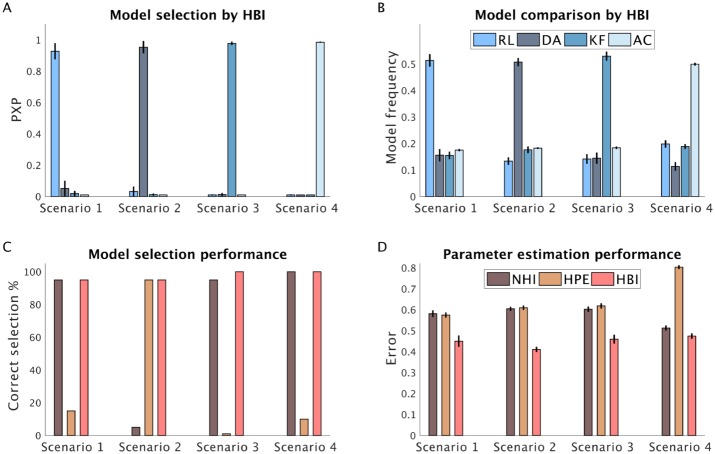
Performance of the HBI in a large model space. HBI was tested in a large model space including RL, dual-*α* (DA) RL, Kalman filter (KF) and actor-critic (AC) models in four scenarios. In each scenario, one model (the dominant model) was used to generate 30 subjects. Other models were used to generate 10 subjects. A) Model selection by HBI using protected exceedance probabilities (PXP). B) Model frequencies estimated by the HBI. Note that in each scenario, the model frequency of the dominant model is 0.5 and it is about 0.17 for the other models. C) Model selection performance (at 50%) of NHI, HPE, and HBI. D) Error in estimating individual parameters across both models and parameters. Estimation errors are computed on the normally distributed parameters, defined as the absolute difference between estimated parameters and the true parameters. In all plots, error-bars are standard errors of the mean obtained across 20 simulations.

So far, we have examined the performance of the HBI in relatively small model spaces. Next, we considered another situation in which 60 subjects are generated according to four different learning models. In addition to the RL, the dual-*α* RL and the Kalman filter model used in previous simulations, here we also considered an actor-critic RL model, which is a class of RL models in which different modules are responsible for learning (critic) and action selection (actor). We considered four scenarios in which 30 subjects were generated according to one of the models and 10 subjects were generated according to each of the other three models (Fig 8). These simulations revealed that protected exceedance probability of the most frequent model computed by the HBI is close to 1. Moreover, the HBI estimate of model frequencies matches well with true frequencies. For reasons detailed in previous analyses, unlike the HBI, the HPE and NHI fail to select the true model in three and one sets, respectively. Furthermore, HBI shows smaller errors in parameter estimation than the other two methods.

**Fig 9:**
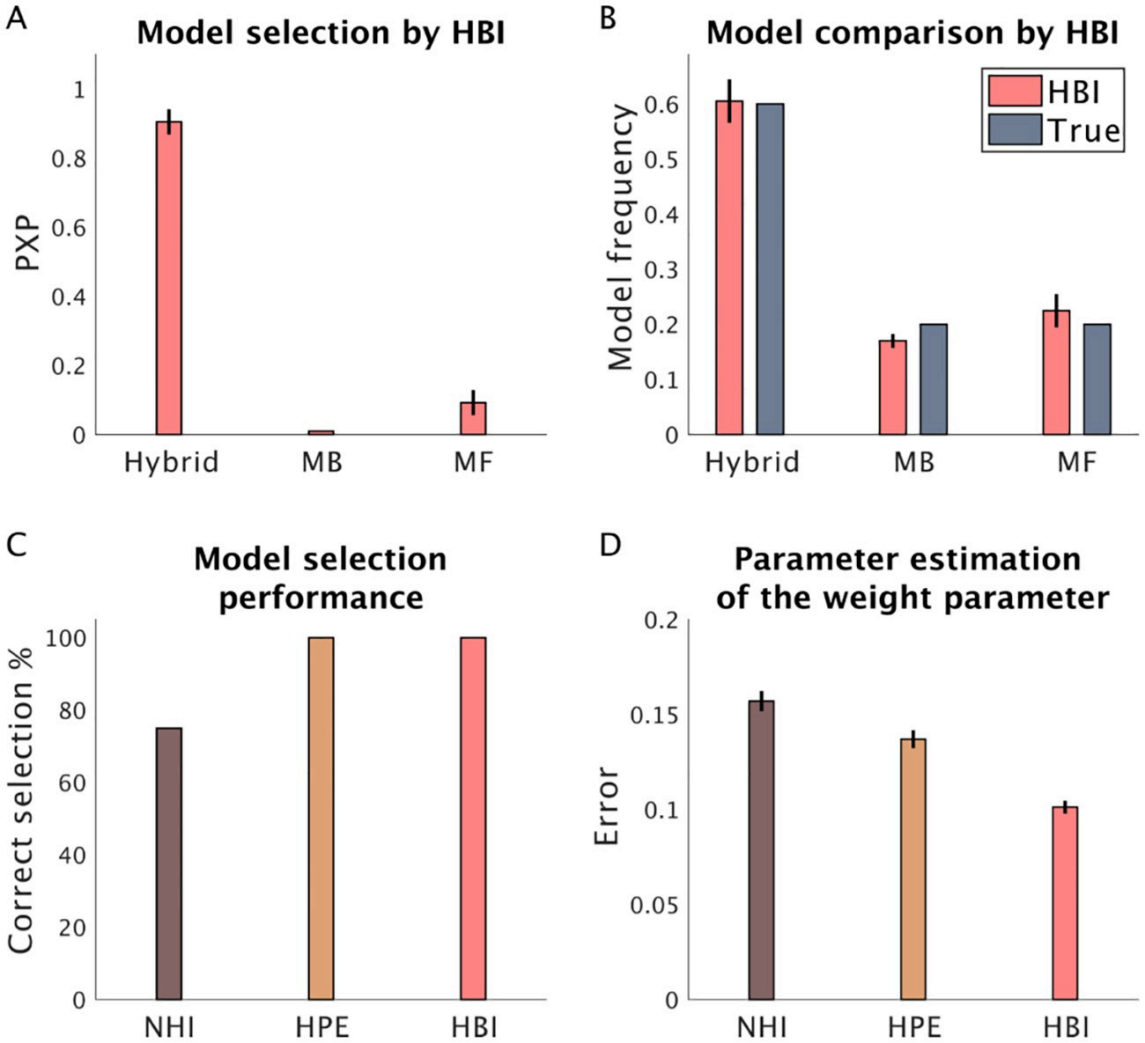
Performance of the HBI in the two-step Markov decision task. 30, 10 and 10 artificial subjects have been generated using the hybrid, the model-based (MB) and the model-free (MF) models, respectively. A) Model selection by HBI using protected exceedance probabilities (PXP). B) Model frequencies estimated by the HBI. C) Model selection performance (at 50%) of NHI, HPE, and HBI. D) Error in estimating the critical weight parameter of the hybrid model at the individual level. HBI shows less error than other methods in all simulations. In all plots, error-bars are standard errors of the mean obtained across 20 simulations.

Finally, we tested the HBI in a more complicated task by considering the two-step Markov decision task introduced by Daw et al. [27]. This task is a well-known paradigm to distinguish two behavioral modes, model-based and model-free learning. Daw et al. [27] have proposed three RL accounts, a model-based, a model-free and their hybrid (which nests the other two and combines their estimates according to a weight parameter), to disentangle the contribution of these two behavioral modes on choices. Here, we skip the details of the models and focus on the application of the HBI to a model space consisting of model-free, model-based and hybrid agents. We generated 30, 10 and 10 artificial subjects according to the hybrid, the model-based and model-free models, respectively (Fig 9). This simulation analysis showed that the HBI performs well in model selection and estimation of model frequencies given true frequencies. Importantly, the HBI recovers the parameters of the models better than alternative methods. In particular, the critical weight parameter of the hybrid model, which determines the degree of balance between the model-based and model-free strategies, was significantly better recovered by the HBI than the other methods (in all 20 simulations, HBI did better than both HPE and NHI).

### HBI t-test for inference at the group-level

#### Sensitivity and specificity of HBI t-test

**Fig 10:**
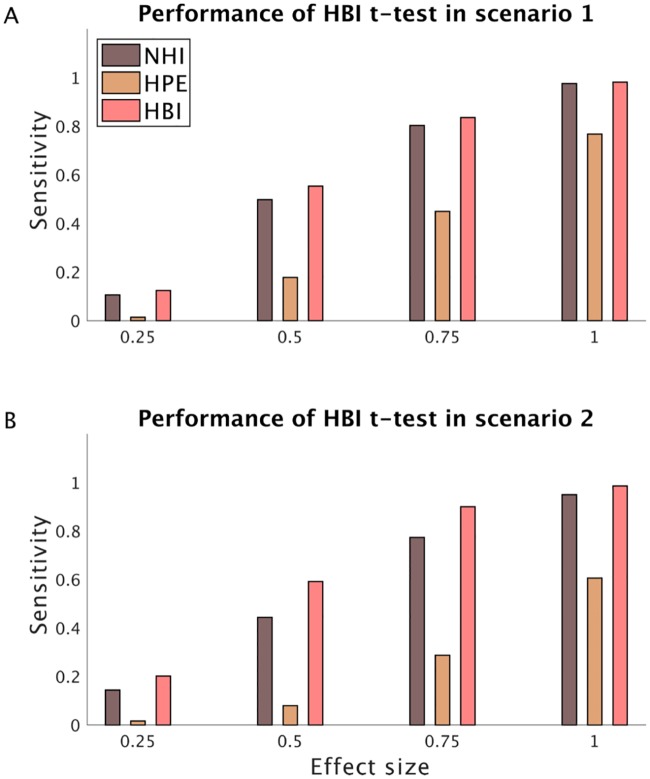
Performance of the HBI t-test for making inference at the population level. RL agents with a bias parameter were generated according to different mean (effect size) values in two simulations where A) there is only one model in the model-space (scenario 1); or B) there are two models in the model-space (scenario 2). The HBI makes inference using the HBI t-test, the NHI makes inference by performing a t-test on its estimated parameters and the HPE makes inference by comparing the full fit and null fit (in which the group-level prior mean for the bias parameter is fixed). The sensitivity (or power) of the tests in detecting true effects at *P* <0.05 for a number of different effect sizes is plotted (i.e. true positive rate). For the HPE, log-evidence of at least 3 was considered as significant. The HPE shows lower sensitivity than the other methods in both scenarios. Moreover, the HBI shows higher sensitivity than the NHI in scenario 2.

We then tested the performance of the HBI t-test introduced above (Fig 10, see Materials and methods for full derivation). In these simulation analyses, we focused on an example that represents a typical inference problem at the population level for parameters of a computational model.

Consider a situation in which subjects should learn stimulus-action-outcome contingencies. The subject’s task is to either to make a go-response by approaching the stimulus or to do nothing (i.e. no-go response). Furthermore, assume that the stimulus is either emotionally appetitive or aversive (e.g. a happy or an angry face cue), but the outcome value is independent of the emotional content of the stimulus. A question of interest is whether the emotional content (happy versus angry) of stimuli induces opposite biases in making a go response, regardless of action values (a form of Pavlovian to instrumental transfer). This is easy to test using an RL model with one additional bias parameter, *b* (we call this model biased RL). The bias is assumed to be +*b* for the emotionally appetitive stimulus and −*b* for the emotionally aversive stimulus. Thus, for larger values of *b*, the subject has a tendency to choose a go response after seeing the emotionally appetitive stimulus and a no-go response after seeing the emotionally aversive stimulus. The bias parameter *b* varies from subject to subject; we are interested here in testing the null hypothesis that its group-level mean is zero.

We simulated a dataset including 20 artificial subjects using this model and a randomly generated reward sequence (binarized Gaussian random-walk). We tested the sensitivity or power of the methods to detect true effects (i.e., nonzero *b*, when present). We repeated this analysis for different effect sizes, in which the bias parameter, *b*, was drawn from a normal distribution with different nonzero effect sizes as its mean, and a variance of 1. A collection of 500 simulations per effect size was simulated. We then compared the performance of HBI in making inference about effects at the group level with that of NHI and HPE. The HBI t-test is very similar to the classical t-test, in which degrees of freedom of the test depends on estimated model frequencies. For NHI, the inference can be done using a classical t-test, as unlike HBI and HPE, samples are treated independently by the NHI. For HPE, one can make inference using Bayesian model selection between a full HPE fit, in which all individual parameters are fitted according to the group level statistics, and a null HPE fit in which the group-level mean and variance for the bias parameter are fixed at their prior value. Note that the group mean of the bias parameter in the null HPE was fixed at zero.

For each simulation analysis, we then quantified accuracy using the HBI t-test at *P* <0.05. Similarly, we quantified the sensitivity of the NHI at *P* <0.05. For the HPE, the log-evidence of at least 3 in favor of the full HPE was defined as a significant effect (which means evidence in favor of the null hypothesis is about 0.05 times less than the alternative hypothesis). These analyses showed that both the HBI and NHI performed quite well in detecting group effects (Fig 10A). The HPE, however, showed low sensitivity. This is because the HPE uses Bayesian information criterion (BIC) to penalize parameters at the group level, which is known to be a conservative metric [23]. Therefore, the full fit HPE loses against the null fit in this stimulation.

We found similar results in another scenario in which samples were generated according to two different models (Fig 10B). Here, the HBI first infers model frequency and then quantifies hierarchical errors and degrees of freedom according to those frequencies. Therefore, we considered the same stimulus-action-outcome learning experiment as above and simulated a dataset including 40 artificial subjects. Data for half of the subjects were generated using the same biased RL model and data for the other half were generated using the dual- RL model explained in previous simulations. Using the same procedure as above, we compared the performance of the HBI, NHI and HPE and found very similar results.

#### HBI t-test under the null hypothesis

Next, we conducted a complementary test of how the HBI t-test performs at avoiding false positives when there are no true effects to be found. Specifically, we tested the performance of HBI t-test for data generated under the null hypothesis, i.e. when the group level mean for the parameter is zero. Note that individual subjects still show a positive or negative bias. Under the null, the p-value generated by the HBI t-test should be uniformly distributed. For example, if null is true, the probability that the p-value falls under 0.05 (the false positive rate) should be 0.05 and the probability that the p-value falls under 0.1 should be 0.1.

**Fig 11:**
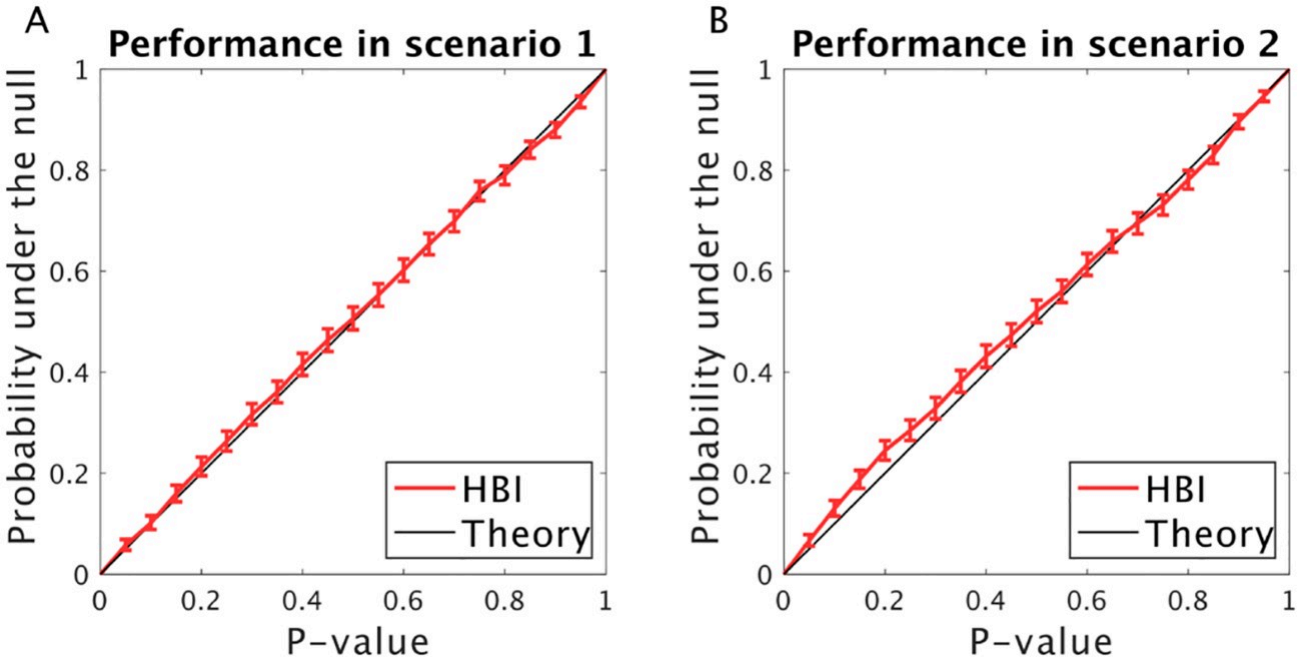
Performance of the HBI t-test under the null. A bias parameter was generated under the null (effect size is 0) in two simulations where A) there is only one model in the model-space (scenario 1); or B) there are two models in the model-space (scenario 2). The probability distribution of P-value is obtained by repeating the simulation 2000 times. Note that under the null hypothesis, the resulting P-value is theoretically expected to have a uniform distribution. The error-bars are 95% confidence intervals for the binomial distribution.

We tested the HBI t-test using the same biased RL model as in previous analyses (Fig 11). The null hypothesis was true here, which means that the individual bias parameters were drawn from a normal distribution with zero mean and variance of 1. We performed 2000 simulations, which allows us to estimate the distribution of p-values generated by the HBI t-test. We found that those p-values are very close to the theoretical uniform distribution (Fig 11A).

We then considered a more difficult scenario in which there are two models in the model space (as above, the biased RL model alongside the dual RL model; Fig 11B). Here, the p-value computed by the HBI t-test depends on the estimated model frequency and even a tiny bias towards one model deteriorates the HBI t-test. Although the performance of the HBI t-test slightly dropped in this scenario, the distribution of p-values was still reasonably good.

#### HBI t-test for skewed samples

**Fig 12:**
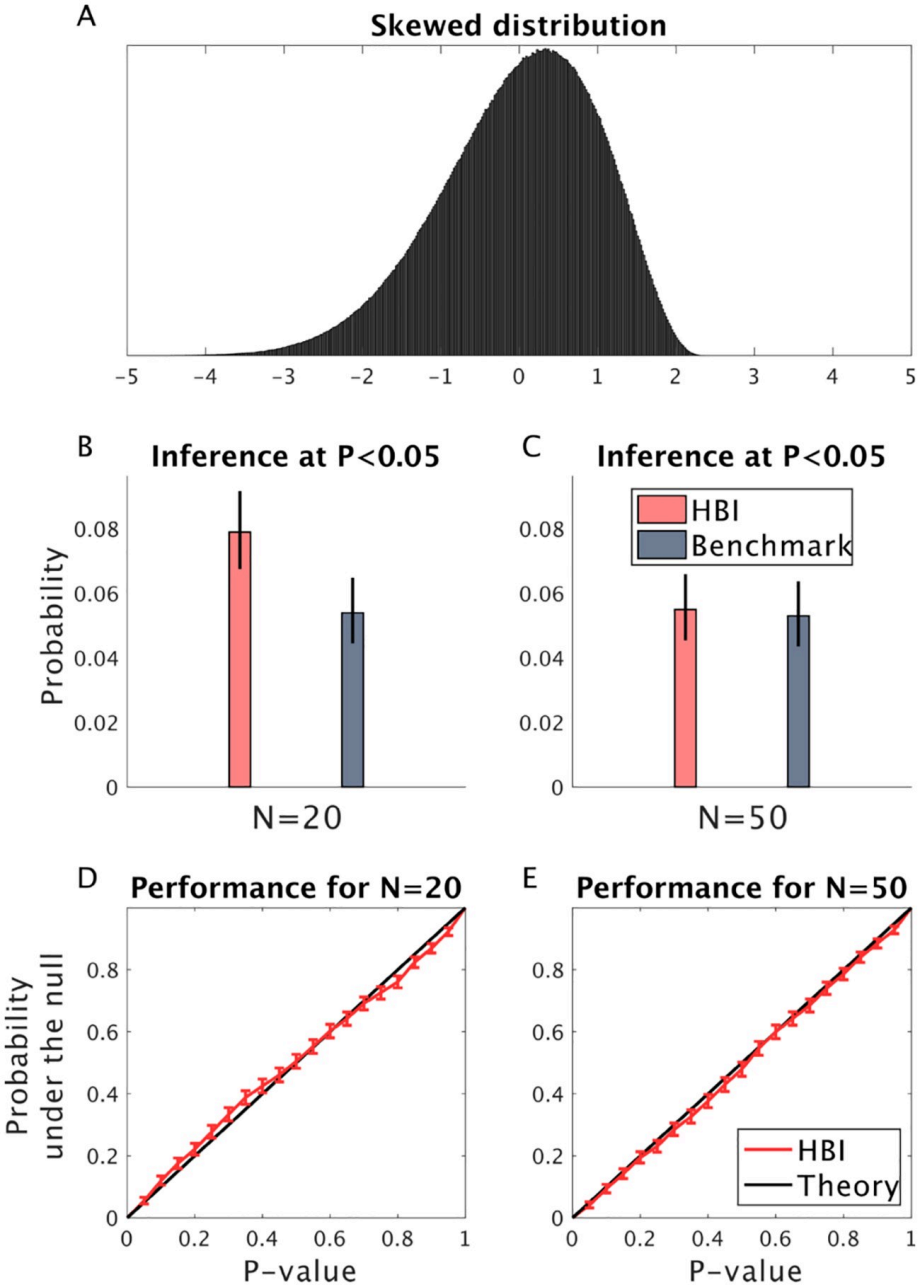
Performance of the HBI t-test when samples are drawn from a skewed distribution. A) The skewed distribution (skewness of −0.5). The mean, variance and kurtosis of the distributions are 0, 1 and 3 (i.e. kurtosis of the normal distribution), respectively. This distribution was used to generate the bias parameter, which was then used to generate 20 (A) and 50 (B) subjects according to the biased RL model. B-C) Inference at *P* <0.05 for the HBI t-test on estimated parameters and t-test on true parameters, as a benchmark, when there is no effect (under the null). Note that this is an unrealistic benchmark because it is based on true parameters that the HBI does not have access to. D-E) Probability of P-value is obtained under the null hypothesis by repeating simulations 2000 times. Under the null hypothesis, the resulting P-value is theoretically expected to have a uniform distribution. Increasing the number of subjects improves the performance of the HBI t-test. The error-bars are 95% confidence intervals for the binomial distribution.

It is well known that the classical t-test is biased when data is generated by a skewed distribution rather than a normal distribution. Since the HBI t-test developed here is also based on a normality assumption, we examined to what extent its performance drops when samples are drawn from a skewed distribution (Fig 12).

We considered the same scenario as in previous simulations, testing false positives in which 20 subjects are generated with the biased RL model. Here, the bias parameter was drawn under the null hypothesis (in the sense that parameter had zero mean, and 1 variance, across subjects), but distributed according to a skewed distribution (with a skewness of –0.5) (Fig 12A). This simulation was repeated 2000 times. First, we compared the probability of finding a significant effect (*P* <0.05) under the null and compared it with the benchmark t-test on true parameters (Fig 12B). Note that this is an unrealistic benchmark as it sees the true parameters. Nevertheless, we found that both tests show statistical biases (as expected theoretically). In particular, both tests showed elevated false positive rates for tests nominally at *P* <.05: false positives occurred for the benchmark test on true parameters was 0.054 and for the HBI t-test on estimated parameters was 0.079. Importantly, increasing the number of samples substantially improves the performance of the HBI t-test as it improves parameter estimation. To investigate this point experimentally, we repeated the same simulation analysis with 50 samples in each dataset. In this simulation, the false positive rate for the benchmark test and the HBI t-test was 0.053 and 0.055, respectively (Fig 12C). Next, we considered the full probability distribution of the p-values under the null hypothesis. As Fig 12D and 12E shows, the mismatch between the estimated and theoretical probabilities reduced by increasing the number of samples.

### Applying HBI to empirical data

**Fig 13:**
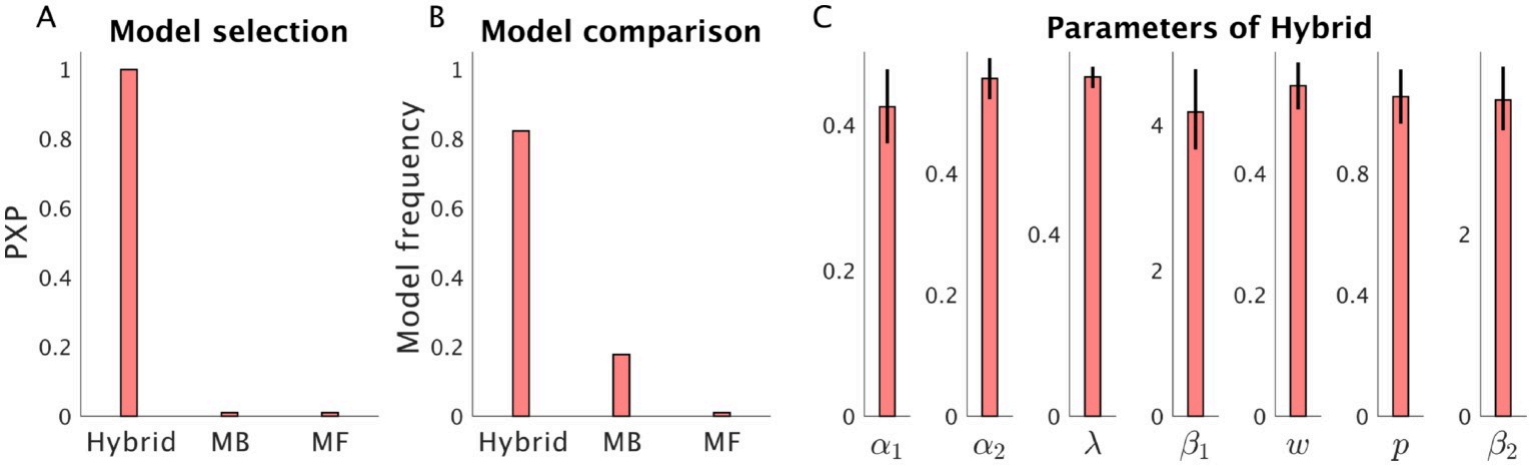
Using HBI for making inference on empirical datasets. A) HBI has been applied to a dataset of the two-step Markov decision task. The model space consisted of the hybrid, the model-based (MB) and the model-free (MF) models. Protected exceedance probabilities (PXP), model frequencies and estimated parameters of the winning model (the hybrid) are plotted. The error-bars are obtained by applying the corresponding transformation function on the hierarchical errors and, therefore, are not necessarily symmetric.

We then applied the HBI method to an empirical choice dataset from 31 subjects performing the two-step Markov decision task. The data used for this analysis have been reported elsewhere [28]. In Fig 13, we have plotted protected exceedance probabilities of each model, model frequencies and estimated group means and corresponding hierarchical errors. According to this analysis, the hybrid model is the most likely model across the group.

We also performed further analysis testing whether individual differences found by the HBI generalize to individual differences in conceptually related, yet independent, data. We reasoned that subjects showing a hybrid strategy might be slower in their choice, as the hybrid model requires combining of model-based and model-free values (which in some trials might be in conflict). Therefore, we looked at the median of response time across all first-level choices for each subject and tested whether there is a difference in response times between those subjects who (according to the separate analysis of choices) employed a hybrid strategy vs. those who employed a model-based strategy as estimated by the HBI. The subgroup attributed to the hybrid model by the HBI showed slower response time compared to those subjects attributed to the model-based account (P = 0.03, Wilcoxon test). These results suggest that HBI reveals meaningful individual differences generalizing to unseen data.

We applied the HBI to another choice dataset of Parkinson’s disease (PD) patients (N = 31), who performed a probabilistic reward and punishment learning task with binary choices (160 trials), which has been used previously for studying maladaptive learning in PD patients. All patients tested on medication. The dataset used here has been reported elsewhere [15].

**Fig 14:**
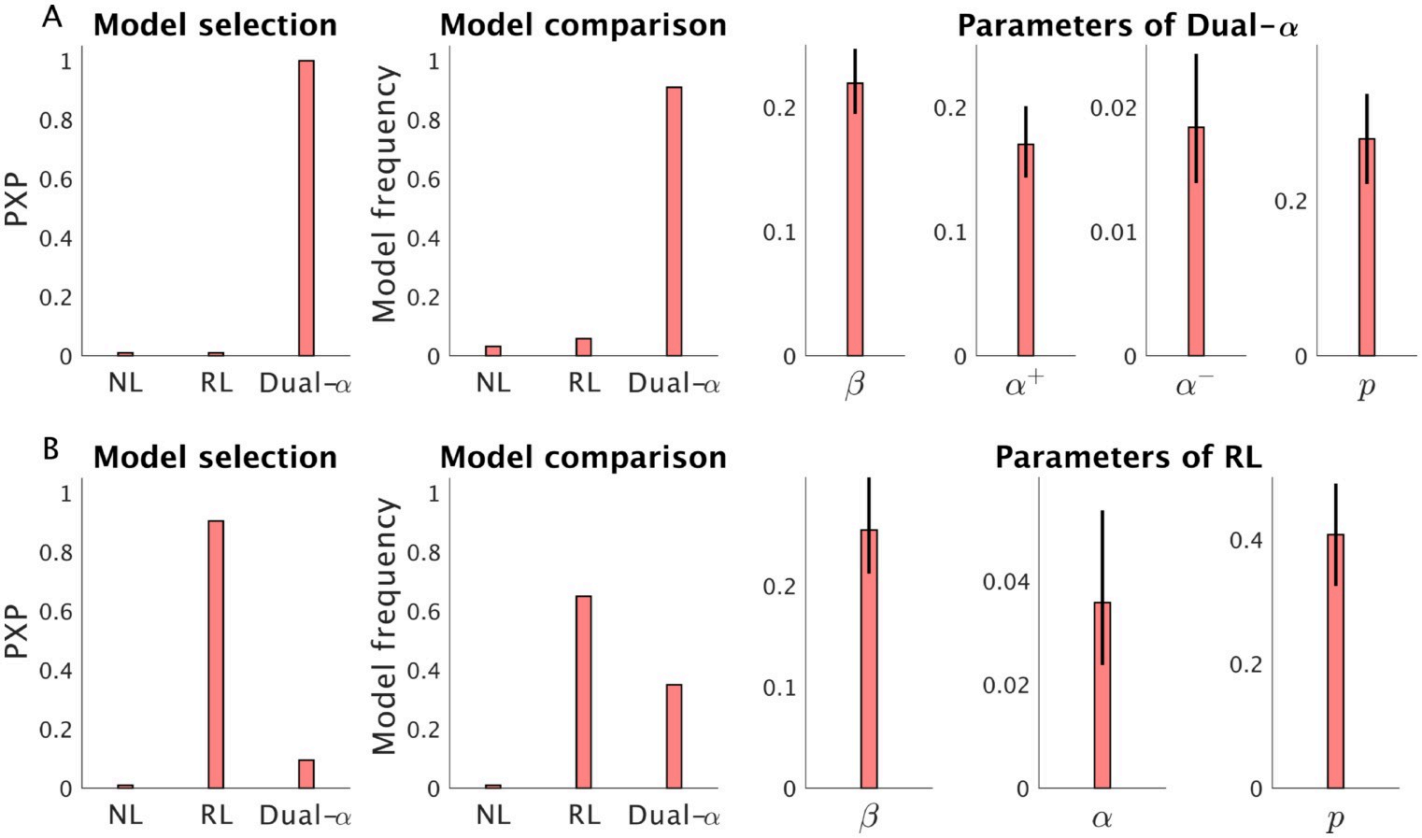
Using HBI for making inference on Parkinson’s patients data. A) HBI has been applied to a dataset of 31 PD patients performing a probabilistic reward and punishment learning task. The model space consisted of a null non-learning (NL) model, RL, and the dual-*α* RL. Protected exceedance probabilities (PXP), model frequencies and estimated parameters of the winning model (the dual-*α* RL) are plotted. The HBI revealed that the dual-*α* RL is more likely across PD patients. B) The same model space was fitted to a dataset of 20 healthy control subjects performing the same task. In contrast to PD patients, the RL model is more likely across the control group. In addition to the decision noise, *β*, and learning rate parameters, both RL models also modeled tendency to repeat or avoid the previous choice regardless of outcomes using a perseveration parameter, *p*. A permutation test revealed that the dual-*α* model is more likely than the RL model in PD compared with the controls. The error-bars are obtained by applying the corresponding transformation function on the hierarchical errors and, therefore, are not necessarily symmetric.

Previous studies proposed that positive and negative prediction errors might be communicated through different dopaminergic receptors or striatal pathways [25, 26, 29], and thus the PD patients might have different learning rate parameters for learning from positive and negative prediction errors [29]. Therefore, we considered a model space including the RL model, the dual- RL model and a simple strategy that selects actions based on the most recent outcome. In both RL models, we also included a perseveration parameter, which models the tendency to repeat or avoid the same choice regardless of the value [15, 30]. This analysis showed that the dual-*α* RL model was more likely across the group. Protected exceedance probabilities, model frequencies and estimated group means and corresponding hierarchical errors are plotted in Fig 14A. We then considered data from matched control participants (N = 20), who performed the same task. The analysis with the HBI showed that the RL model is more likely for the control group (Fig 14B), suggesting that PD (or dopaminergic medication in PD) increases the discrepancy between the learning rates for positive and negative prediction errors. We finally performed a permutation test to formally test the significance of this difference (1000 permutations). For each permutation, all participants were randomly divided into control and PD groups with the same size as the real control and PD groups. The HBI was then used to fit the same model space to each random group. The relative model frequency statistics (RL vs. dual-*α* RL) was quantified for each permutation. This permutation test confirmed that the dual-*α* RL was significantly more likely than the RL model in PD patients compared with controls (*P* <0.001).

## Discussion

In this work, we have introduced a novel method, a hierarchical and Bayesian inference framework, for parameter estimation and model comparison. The HBI framework is hierarchical in the sense that parameters at the individual level are regularized by statistics across all individuals in the group. The HBI framework is Bayesian in the sense that all uncertainties at both individual and group levels are represented by probability distributions. The HBI framework has major theoretical advantages over current state-of-the-art methods, mainly because it combines, in a single hierarchical structure, two sorts of inference (about model identity and model parameters), which are interdependent but have previously been treated separately. Our simulation results demonstrated these advantages experimentally.

In this work, we took an empirical Bayes approach [31, 32], in which priors are constructed based on data. In other words, parameters at the individual level are regularized by statistics across all individuals in the group. Furthermore, we took a so-called random effects approach to model identity [13], which indicates that different models might underlie data in different subjects. This is in contrast to previous hierarchical methods for model fitting, which assume the same model underlie data in all subjects (fixed effects assumption [10, 11]). The random effects approach to hierarchical inference has important consequences for both parameter estimation and model comparison. Moreover, we took a fully Bayesian approach by quantifying uncertainty at the group level, which enabled us to develop statistical tests about group parameters and to quantify corresponding statistical errors.

Empirical Bayes methods play an increasing role in modern statistics. These methods essentially take a hierarchical approach, by assuming that individual data are generated based on the probabilistic properties of the population. This hierarchical approach has important consequences. The most important consequence is that they provide a promising solution to the classical problem of priors in Bayesian statistics by providing informative, yet objective, priors at the individual level. Furthermore, by partly sharing parameters across subjects, they reduce overfitting relative to non-hierarchical models, which in turn allows them to confidently fit more complex models with a smaller penalty for overfitting. This is because non-hierarchical methods assume that the extra parameters of a complex model are independent. For example, consider a model space in which the more complex model has one extra free parameter and there are 40 subjects in the dataset. Fitting the dataset with the complex model using non-hierarchical methods introduces 40 additional independent free parameters, driving the danger of overfitting, and accordingly an excessive penalty to account for this possibility in assessing the evidence for the model. The hierarchical approach, however, assumes that the individual parameters are dependent, as they are all generated according to the same distribution, sharing a single mean parameter and smaller deviations from it. Modeling this hierarchical dependency enables those methods to avoid penalizing complex models as excessively. Our simulation results demonstrate this point experimentally (Fig 3D). While the non-hierarchical method failed to select the correct model with one additional parameter, evidently because the overfitting penalty was too extreme, the HBI was successful in selecting the correct model (Fig 3D).

The HBI method introduced in this paper is built based on the random effects view that different models might underlie data in different subjects. Taking this view enabled us to address problems caused by taking the model identity as a fixed effect in some hierarchical parameter estimation procedures. For parameter estimation, the fixed effects assumption biases the group parameters because it assumes that all subjects contribute equally to the group parameters. The proposed HBI framework solves this problem by weighting contribution of each subject to group statistics by the degree to which that model is likely to be the true underlying model for that subject (Figs 1 and 3). For model comparison, the fixed effects assumption leads to oversensitivity to outliers as the evidence across the group is driven by the sum of individual evidence. Our simulation results (Fig 2) showed that only a few outliers lead to incorrect model selection inference made by the fixed effects assumption. The proposed HBI method solves this problem by normalizing individual evidence across all candidate models. Specifically, the HBI framework quantifies the responsibility of each model *k* in generating each subject data, a metric lying between 0 and 1. For every subject, the responsibility sums up to 1 across all candidate models as it partitions probability space among those models (see [13, 19] for a similar non-hierarchical approach). It is then easy to compare models by enumerating responsibilities across the group in favor of each model or by estimating the most likely model.

Another major contribution of this paper is to provide a statistical test, HBI t-test, to the inference problem at the group level using hierarchically fitted parameters. For models fitted by a non-hierarchical method, such as maximum likelihood or Laplace approximation, it is statistically valid to use classical statistical tests on fitted parameters to make inference at the group level. However, for datasets fitted by a hierarchical method in which the individual fits are regularized according to statistics of the group data, conventional statistical tests are not valid, because the parameter estimates are non-independent from subject to subject. Our fully Bayesian approach enabled us to address this issue. Our method provides an intuitive solution to this problem in the form of a t-statistic, in which all the group statistics are computed according to the estimated responsibilities of the corresponding model in generating each individual data. Thus, the HBI quantifies the uncertainty of the group parameters and thereby the corresponding hierarchical errors. Our analysis showed that the HBI performed better than both the NHI and HPE in detecting true effects and also that it was well calibrated, displaying the appropriate number of false positives when effects were absent. Therefore, the HBI framework enables researchers to make statistical claims about parameters at the group level.

It is important, however, to note that the foundation of the HBI t-test is completely different from the classical t-test, as it is a Bayesian (in contrast to frequentist) test using posterior probabilities. In particular, this test is based on the posterior distribution of the statistics of interest (i.e. group mean) marginalized over all other parameters (e.g. group variance), which is given by a Student’s t-distribution (Eq 24). Statistically, the precise claim of the HBI t-test is that whether a specific point is outside of a credible interval, which is the interval that the group parameter value falls with a particular subjective probability. For example, if the HBI t-test indicates that a parameter is significantly different from 0 at *P* <0.05, it means that 0 does not fall within the 95% credible interval. One important difference between Bayesian credible intervals and classical (frequentist) confidence intervals (used in classical Student’s t-test) is that Bayesian credible intervals depend on priors. However, since we used minimally-informative priors (statistically proper priors with very little effects on posteriors, see Materials and methods), the HBI t-test almost entirely depends on data. In fact, that is the reason that under the null, the HBI t-test generates p-values uniformly as shown by simulations (Fig 11). Notably, the same Student distribution can also be used to accept the null hypothesis for example using a “region of practical equivalence” procedure described by Kruschke [33]. It is also possible to employ the more common way and make inference in favor of the null hypothesis using model selection. In this case, one needs to perform a model selection between a model in which the group-level mean of the parameter of interest is fixed at the null value (null model) and compare that with a full HBI with no restriction (alternative model) using Bayes factor (i.e. difference log model evidence).

In addition to model comparison, the HBI framework can also be used for model selection in situations where the goal is to select one of the models as the best model across the group. Exceedance probability is a metric proposed [13] to perform model selection using a random effects approach. An important revision of this metric called protected exceedance probability [19] also takes into account the null possibility that none of the models in model space is supported sufficiently by data, i.e. the differences in model evidence are due to chance. As the HBI framework treats model identity as a random effect, it is possible to compute exceedance and protected exceedance probabilities (Eqs 26–28). Note that if this procedure indicates that models’ ability to explain data are not different (i.e. their difference is likely to be due to chance), one cannot rely on estimated parameters, as those are also dependent on estimated model frequencies. In this situation, we recommend to obtain parameters by fitting models separately to data using the HBI, which makes sense as there is no evidence that models are differently expressed across subjects. In our analysis with simulated and empirical data, however, we never encountered this situation as probability of the null (*P*_0_ in Eq 28) was always very small.

In this study, we compared the performance of the HBI with two alternative methods with different statistical assumptions about the generative process of data. The NHI assumes a hierarchy in model identity for generating individual data. The HPE assumes that parameters are generated in a hierarchical fashion, but assumes no hierarchy regarding model identities. The HBI assumes that both model identity and parameters are generated hierarchically. Importantly, the inference procedure for all these methods is very similar, which allows a fair comparison of them largely based on their statistical assumptions. In particular, the three methods all employ Laplace approximation for making a quadratic approximation of individual-level posteriors. Furthermore, the HBI is based on variational Bayes, which is an extension to the case of multiple latent variables of the expectation-maximization procedure used previously for implementing the HPE [10, 11] (see also [34] for a variational implementation), which itself extends the one-level Bayesian inference of NHI. There are other ways to make an inference, for example using Markov chain Monte Carlo methods. Future studies should investigate the pros and cons of those methods, compared with the variational Bayes used here, for making inference in HBI.

There are increasing efforts to exploit advances in computational modeling for understanding mental disorders [3–6]. Recent works, however, have started to tackle challenges related to quantifying uncertainty in diagnosis and also in the evaluation of treatment effects. For example, hierarchical unsupervised generative modeling, have used Monte-Carlo and variational methods to identify a cluster of subjects showing similar patterns of neural connectivity [35, 36]. HBI also offers a promising solution by quantifying uncertainty in model attribution to individuals. Our simulation analyses showed that the HBI performs better than other alternatives in model attribution. This can help us to move towards better diagnosis and precise evaluation of different treatments [37].

In summary, the HBI framework proposed in this work rests on a hierarchical view of both hypothesis testing (i.e. model comparison) and parameter estimation for multi-subject studies and thus provides a generic framework for statistical inference. Moreover, the HBI framework runs fully automatically and it does not rely on hand tuning of parameters. Therefore, we expect this method to be useful for a wide range of studies testing different hypotheses in a multi-subject setting. This includes not only computational models of learning and decision making but also any statistical models of brain or behavior.

## Materials and methods

Here, we give a formal treatment of the HBI framework in seven sections, in which we 1) define the probabilistic model underlying HBI; 2) lay out the basis of our variational approach for making inference (the full proof is given in S1 Appendix); 3) present the HBI algorithm; 4) derive the HBI t-test; 5) show how HBI can be used for making inference about a new subject; 6) define important practical points, in particular prior parameters, initialization and convergence criteria; 7) give a formal definition of the exceedance and protected exceedance probability. The HBI and its manual are freely available online as part of computational and behavioral modeling (cbm) toolbox: https://payampiray.github.io/cbm.html.

### Probabilistic model

We begin by describing the probabilistic model of the HBI. Consider an observed dataset **X** = {**x**_1_, …, **x**_*N*_} where **x**_*n*_ is the dataset (e.g. choices) of *n*th subject and *N* indicates the number of subjects and a model-space including *K* candidate models, *M*_1_…*M*_*K*_. Moreover, suppose that the prior probability of each model in the population is given by **m** = {*m*_1_, …, *m*_*K*_}. For each dataset, **x**_*n*_, we assume that there is a latent variable **z**_*n*_ comprising a 1-of-K binary random vector, in which *z*_*kn*_ is one if **x**_*n*_ generated is by the *k*th model. Thus, the probability of the latent variable across all subjects, **Z** = {**z**_1_, …, **z**_*N*_}, is assumed to have a multinomial distribution,

$$\begin{array}{c} p\left(Z|m\right)=\prod_{n}\prod_{k}m_{k}^{z_{kn}}. \end{array}$$ (1)

Each model *M*_*k*_ in the model-space is supposed to compute the probability of a given dataset (e.g. a set of choices) given a set of parameters, **h**_*kn*_. For example, the reinforcement learning model computes the probability of choices using two parameters: a learning rate and a decision noise parameter. The number of models and their structures depend on specific scientific questions. Here, we take a general approach by making no specific assumption about the number of models, *K*. Thus, the *k*th model in the model-space, *M*_*k*_, computes the probability of dataset **x**_*n*_ given the parameter vector **h**_*kn*_, which is denoted by *p*(**x**_*n*_|**h**_*kn*_, *M*_*k*_). Note that the number of parameters in model *k*, denoted by *D*_*k*_, might be different across models. Since data for each subject is generated by one of the models, which is denoted in the binary vector **z**_*n*_, the probability of the observed dataset given the model-space is

$$\begin{array}{c} p\left(X|H,Z\right)=\prod_{k}\prod_{n}p{\left(x_{n}|h_{kn},M_{k}\right)}^{z_{kn}}, \end{array}$$ (2)

where **H** denotes all the parameters across all participants and models. The parameters of *k*th model are assumed to have a multivariate normal distribution with mean ***μ***_*k*_ and precision matrix **T**_*k*_,

$$\begin{array}{c} p\left(H|Z,\mu ,T\right)=\prod_{k}\prod_{n}N{\left(h_{kn}|\mu_{k},T_{k}^{-1}\right)}^{z_{kn}}, \end{array}$$ (3)

where **T**_*k*_ is a diagonal matrix with positive elements.

We also introduce a distribution over model frequencies, **m**. We use the Dirichlet distribution, which forms the conjugate prior for the multinomial distribution, as the prior:

$$\begin{array}{c} p\left(m\right)=\text{Dir}(m|\alpha_{0})=C\left(\alpha_{0}\right)\prod_{k=1}^{K}m_{k}^{\alpha_{0}-1}, \end{array}$$ (4)

where *C*(*α*_0_) is the normalizing constant for the Dirichlet distribution.

We also take group parameters ***μ*** and **T** as random variables, which allows us to evaluate their posterior distribution given data. We introduce conjugate priors for these variables, a Gaussian-Gamma prior in which the distribution over ***μ***_*k*_ depends on **T**_*k*_:

$$\begin{array}{c} p\left(\mu |T\right)=\prod_{k=1}^{K}N\left(\mu_{k}|a_{0},{\left(bT_{k}\right)}^{-1}\right) \end{array}$$

$$\begin{array}{c} p(T)=\prod_{k=1}^{K}\prod_{i=1}^{D_{k}}G\left(\tau_{ki}|v,s\right), \end{array}$$

where G(.) denotes Gamma distribution. Here, *τ*_*ki*_ is the *i*th diagonal element of **T**_*k*_. Assuming that ***τ***_*k*_ is a vector containing *τ*_*ki*_, by defining **T**_*k*_ = diag(***τ***_*k*_), in which diag(.) is an operator outputting a diagonal matrix with elements given by ***τ***_*k*_, we can write these two equations in a compact form:

$$\begin{array}{c} p\left(\mu ,\tau \right)=\prod_{k=1}^{K}N\left(\mu_{k}|a_{0},\text{diag}{\left(b\tau_{k}\right)}^{-1}\right)G\left(\tau_{k}|v,s\right), \end{array}$$ (5)

where we have defined:

$$\begin{array}{c} G\left(\tau_{k}|v,s\right)=\prod_{i=1}^{D_{k}}G\left(\tau_{ki}|v,s\right), \end{array}$$

in which *v* is a scalar and **s** is a vector with *D*_*k*_ elements all equal to *s*. The full probabilistic model is given by,

$$\begin{array}{c} p(X,H,Z,\mu ,\tau ,m)=p(X|H,Z)p(H|Z,\mu ,\tau )p(Z|m)p(\mu |\tau )p(\tau )p(m). \end{array}$$ (6)

### Variational inference

The task of Bayesian inference is to compute the posterior probabilities of latent variables given data, *p*(**H**, **Z**, ***μ***, ***τ***, **m**|**X**). Since the inference is intractable for the probabilistic model outlined in the previous section, we employ variational inference to compute approximate posteriors. We take a so-called mean-field approach [16, 17] by assuming that the posterior is partially factorized as follows:

$$\begin{array}{c} q(H,Z,\mu ,\tau ,m)=q(H,Z)q(\mu ,\tau ,m). \end{array}$$ (7)

Note that we force no factorization in the posterior between latent variables, **Z** and **H**. Using a quadratic approximation of the conditional posterior, *q*(**H**|**Z**), we prove in S1 Appendix that these posteriors are given by,

$$\begin{array}{c} q\left(H,Z\right)=\prod_{k}\prod_{n}r_{kn}^{z_{kn}}N{\left(h_{kn}|\theta_{kn},A_{kn}^{-1}\right)}^{z_{kn}} \end{array}$$ (8)

$$\begin{array}{c} q\left(\mu ,\tau ,m\right)=\text{Dir}\left(m|\alpha \right)\prod_{k}q\left(\mu_{k},\tau_{k}\right) \end{array}$$ (9)

$$\begin{array}{c} q\left(\mu_{k},\tau_{k}\right)=N\left(\mu_{k}|a_{k},\text{diag}{\left(\beta_{k}\tau_{k}\right)}^{-1}\right)G\left(\tau_{k}|\nu_{k},\sigma_{k}\right), \end{array}$$ (10)

where 0 ≤ *r*_*kn*_ ≤ 1 is the responsibility of model *k* for *n*th subject, ***θ***_*kn*_ and **A**_*kn*_ are the subject-level mean and precision, *ν*_*k*_ and *β*_*k*_ are scalars and ***σ***_*k*_ is a vector with the same size as ***τ***_*k*_. In the next section, we provide the HBI algorithm, which iteratively updates the parameters of these distributions, *r*_*kn*_, ***θ***_*kn*_, **A**_*kn*_, ***α***, **a**_*k*_, *ν*_*k*_, *β*_*k*_, and ***σ***_*k*_.

### HBI algorithm

After initializing the individual parameter estimates, ***θ***_*kn*_ and **A**_*kn*_ and responsibilities *r*_*kn*_ for all subjects and models, as well as setting prior parameters **a**_0_, *b*, *s*, *v* and *α*_0_ (which will be defined later), the HBI algorithm performs these steps:

1. Calculate the summary statistics:
  $$\begin{array}{c} \bar{N}{\;}_{k}=\sum_{n}r_{kn} \end{array}$$ (11)
  $$\begin{array}{c} \;\bar{\theta }{\;}_{k}=\frac{1}{{\bar{N}}_{k}}\sum_{n}r_{kn}\theta_{kn} \end{array}$$ (12)
  $$\begin{array}{c} \;\bar{V}{\;}_{k}=\frac{1}{{\bar{N}}_{k}}\sum_{n}r_{kn}(\theta_{kn}\theta_{kn}^{⊤}-\;\bar{\theta }{\;}_{k}\;\bar{\theta }{\;}_{k}^{⊤}+A_{kn}^{-1}). \end{array}$$ (13)
2. Update parameters of *q*(***μ***, ***τ***, **m**) for all models:
  $$\begin{array}{c} a_{k}=\frac{1}{\bar{N}{\;}_{k}+b}\left({\bar{N}}_{k}\;\bar{\theta }{\;}_{k}+ba_{0}\right) \end{array}$$ (14)
  $$\begin{array}{c} \beta_{k}=b+{\bar{N}}_{k} \end{array}$$ (15)
  $$\begin{array}{c} \sigma_{k}=s+\frac{1}{2}\text{diag}(\bar{N}{\;}_{k}\;\bar{V}{\;}_{k}+\frac{b\;\bar{N}{\;}_{k}}{b+\;\bar{N}{\;}_{k}}\left(\;\bar{\theta }{\;}_{k}-a_{0}\right){\left(\;\bar{\theta }{\;}_{k}-a_{0}\right)}^{⊤}) \end{array}$$ (16)
  $$\begin{array}{c} \nu_{k}=v+\frac{1}{2}{\bar{N}}_{k} \end{array}$$ (17)
  $$\begin{array}{c} \alpha_{k}=\alpha_{0}+{\bar{N}}_{k}. \end{array}$$ (18)
3. Update the individual posterior parameters ***θ***_*kn*_, **A**_*kn*_ and *f*_*kn*_, by obtaining a quadratic approximation of the function, *ℓ*_*kn*_(**h**), with respect to **h**:
  $$\begin{array}{c} \ell_{kn}\left(h\right)=p\left(x_{n}|h,M_{k}\right)N\left(h|E\left[\mu_{k}\right],E{\left[T_{k}\right]}^{-1}\right), \end{array}$$ (19)
  where $E\left[\mu_{k}\right]=a_{k}$ and $E{\left[T_{k}\right]}^{-1}=\frac{1}{\nu_{k}}\text{diag}\left(\sigma_{k}\right)$. This approximation can be written as
  $$\begin{array}{c} \ell_{kn}\left(h\right)≃f_{kn}\exp\left(-\frac{1}{2}{\left(h_{kn}-\theta_{kn}\right)}^{⊤}A_{kn}\left(h_{kn}-\theta_{kn}\right)\right). \end{array}$$ (20)
  Note that any quadratic approximation can be used here. For example, using a Laplace quadratic approximation (which is a very common approximation for analyzing behavioral and neural data [20, 22, 23]), ***θ***_*kn*_, **A**_*kn*_ and *f*_*kn*_ are given by the mode, Hessian of log *ℓ*_*kn*_ and the maximum value of *ℓ*_*kn*_, respectively:
  $$\begin{array}{c} \theta_{kn}=\arg\max_{h}\text{log}\ell_{kn}\left(h\right) \end{array}$$
  $$\begin{array}{c} A_{kn}=-\nabla \nabla \text{log}\;\ell_{kn}{\left(h\right)|}_{\theta_{kn}} \end{array}$$
  $$\begin{array}{c} f_{kn}=\ell_{kn}\left(\theta_{kn}\right). \end{array}$$
4. Update responsibilities,
  $$\begin{array}{c} r_{kn}=\frac{\rho_{kn}}{\sum_{j=1}^{K}\rho_{jn}}, \end{array}$$ (21)
  where
  $$\begin{array}{c} \text{log}\;\rho_{kn}=\text{log}\;f_{kn}+\frac{1}{2}D_{k}\;\text{log}\;2\pi -\frac{1}{2}\text{log}\;\left|A_{kn}\right|+\lambda_{k}+E\left[\text{log}\;m_{k}\right] \end{array}$$ (22)
  $$\begin{array}{c} \lambda_{k}=\frac{D_{k}}{2}(\psi \left(\nu_{k}\right)-\text{log}\;\nu_{k}-\frac{1}{\beta_{k}}) \end{array}$$ (23)
  $$\begin{array}{c} E\left[\text{log}\;m_{k}\right]=\psi \left(\alpha_{k}\right)-\psi \left(\sum_{k=1}^{K}\alpha_{k}\right), \end{array}$$
  in which *ψ*(.) is the digamma function.
5. Terminate if stopping criteria are met, otherwise go to 1.

### Statistical tests for group parameters

An important goal of computational modeling studies is to compute the distribution of parameters given data across the whole population. From a Bayesian viewpoint, this is given by the marginal posterior over the mean of group parameters, ***μ***_*k*_, which reads

$$\begin{array}{cc} p(\mu_{k}|X) & ≃\int q\left(\mu_{k},\tau_{k}\right)d\tau_{k}\\ & =\int N\left(\mu_{k}|a_{k},{\left(\beta_{k}\tau_{k}\right)}^{-1}\right)G\left(\tau_{k}|\nu_{k},\sigma_{k}\right)d\tau_{k}\\ & =St(\mu_{k}|a_{k},\eta_{k},n_{k}), \end{array}$$

where $n_{k}=2\nu_{k}=2v+{\bar{N}}_{k}$ is the number of degrees of freedom of the Student distribution and $\eta_{k}=\nu_{k}\beta_{k}\sigma_{k}^{-1}$ is the inverse-scale parameter. Therefore, the random variable $t=\eta_{k}^{\frac{1}{2}}\left(\mu_{k}-a_{k}\right)$ takes a form of standard Student distribution with *n*_*k*_ degrees of freedom. By defining $s_{ki}^{2}=\frac{2}{\beta_{k}}\sigma_{ki}$, in which $s_{ki}^{2}$ corresponds to empirical variance (c.f. Eq (16)), we can write this result in an intuitive form,

$$\begin{array}{c} p\left(\mu_{ki}|X\right)=St(\frac{\mu_{ki}-a_{ki}}{s_{ki}/\sqrt{n_{k}}}|n_{k}). \end{array}$$ (24)

Noting the similarity between $s_{ki}/\sqrt{n_{k}}$ and the standard error of the mean, we called $s_{ki}/\sqrt{n_{k}}$ the hierarchical error. Note that if we assume *v* = 0.5 (which is reasonable as explained later), we obtain $n_{k}=1+{\bar{N}}_{k}$.

### Predictive distribution for a new subject

In many situations, researchers are interested to fit a new dataset to a particular model and find corresponding parameters. In Bayesian statistics, this is called the predictive distribution and it is given by marginalizing over group parameters. Suppose that x* and $h_{k}^{*}$ denote the new dataset and its corresponding parameters for model *k*. The marginal distribution $p(x^{*},h_{k}^{*}|z_{k}^{*}=1,X)$ is the predictive distribution given the observed dataset **X** assuming that the new data is generated by the *k*th model. This distribution is given by:

$$\begin{array}{cc} p(x^{*},h_{k}^{*}|z_{k}^{*}=1,X) & =\int p\left(x^{*}|h_{k}^{*},M_{k}\right)p\left(h_{k}^{*}|\mu_{k},\tau_{k},z_{k}^{*}=1\right)p\left(\mu_{k},\tau_{k}|X\right)d\mu_{k}d\tau_{k}\\ & =p\left(x^{*}|h_{k}^{*},M_{k}\right)St\left(h_{k}^{*}|a_{k},{\left(1+\beta_{k}\right)}^{-1}\eta_{k},n_{k}\right), \end{array}$$

where ***η***_*k*_ and *n*_*k*_ have been defined in the previous section. This distribution can also be written in terms of standard Student distribution with *n*_*k*_ degrees of freedom. Furthermore, if we assume that *b* = 2*v*, which is a reasonable assumption (see the next section), this distribution is given by

$$\begin{array}{c} p\left(x^{*},h_{k}^{*}|z_{k}^{*}=1,X\right)=p\left(x^{*}|h_{k}^{*},M_{k}\right)St\left(\text{diag}{\left(s_{k}\right)}^{-1}\left(h_{k}^{*}-a_{k}\right)|n_{k}\right), \end{array}$$

where **s**_*k*_ is a vector of corresponding empirical deviance parameters, defined in the previous section. Using this joint distribution, one can use sampling methods to obtain the posterior over parameters, $p(h_{k}^{*}|z_{kn}=1,X,x^{*})$, or to obtain the maximum-a-posteriori parameters, $\theta_{k}^{*}$, given by

$$\begin{array}{c} \theta_{k}^{*}=\arg\max_{h}\text{log}p\left(x^{*}|h,M_{k}\right)St\left(\text{diag}{\left(s_{k}\right)}^{-1}\left(h-a_{k}\right)|n_{k}\right). \end{array}$$ (25)

Note that for many degrees of freedom due to large values of ${\bar{N}}_{k}$, the Student distribution tends to a Gaussian with mean **a**_*k*_ and deviance matrix diag(**s**_*k*_). However, small values of ${\bar{N}}_{k}$ lead to a small number of degrees of freedom and heavier tailed distributions than Gaussians, which are more robust against outliers.

### Parameters, initialization and convergence criteria

As the mean-field variational inference is an iterative framework, it also depends on the initialization of the parameters. In this section, we provide priors that do not bias the final solution and also provide some intuitive criteria for the initialization.

We initialize the parameters ***θ***_*kn*_ and **A**_*kn*_ by fitting all models separately to all participants (with some initial Gaussian prior), i.e., assuming as if *z*_*kn*_ = 1. These values are then used to calculate summary statistics according to Eqs (11)–(13).

Furthermore, we need to define prior parameters. The free parameter *α*_0_ indicates prior frequency of each model. We take uninformative priors on frequency of models, which is given by *α*_0_ = 1 for all models. The prior mean, **a**_0*k*_, is assumed to be zero. Given Eq (15), we see that *b* can be interpreted as the effective number of prior samples associated with models. Also, given Eq (17), *v* could be interpreted as the half of the effective number of prior samples associated with models. Assuming that the priors account for one sample, which is a common assumption in Bayesian statistics, we take *b* = 1 and $v=\frac{1}{2}$. Finally, since *s* has always an additive effect on ***σ***_*k*_ according to Eq (16), we assume a small positive value for *s*, allowing that ***σ***_*k*_ to be driven dominantly by data. In all our analyses, we assumed *s* = 0.01. It is also important to note that by choosing a small value for *s*, we ensure that if a model loses entirely (takes no responsibility), its corresponding parameters at the individual level converge to the prior mean, **a**_0*k*_, with a very small variance.

Finally, the HBI algorithm presented above requires stopping criteria. In our analyses, we terminated the algorithm if the change in normalized value of parameters between two consecutive iterations, *j* − 1 and *j*, defined as

$$\begin{array}{c} \hat{d}=\sqrt{\frac{1}{K}\sum_{k}\frac{1}{D_{k}}\sum_{i}{\left({\hat{\theta }}_{ki}^{j}-{\hat{\theta }}_{ki}^{j-1}\right)}^{2}}, \end{array}$$

was smaller than 0.01. Here, ${\hat{\theta }}_{ki}^{j}$ is defined according to summary statistics of parameters on the *j*th iteration:

$$\begin{array}{c} {\hat{\theta }}_{ki}^{j}=\;\bar{\theta }{\;}_{ki}/\;\bar{V}{\;}_{ki}^{\frac{1}{2}}, \end{array}$$

where $\;\bar{\theta }{\;}_{ki}$ and $\;\bar{V}{\;}_{ki}$ are the *i*th element of $\;\bar{\theta }{\;}_{k}$ and $\;\bar{V}{\;}_{k}$ defined in (12 and 13), respectively. In our analyses, we also set 50 as the maximum number of iterations, although almost always the algorithm stopped before hitting this number.

### Exceedance probability and protected exceedance probability

Using the posterior over **m**, one can also derive the so-called exceedance probability and protected exceedance probability, as defined in previous works [13, 19]. We reproduce the equations here for completeness.

The exceedance probability of *k*th model, *ϕ*_*k*_, is defined as the probability that model *M*_*k*_ is more likely than any other model in the model-space and it is given by

$$\begin{array}{c} \phi_{k}=\text{Prob}\left(m_{k}>m_{j}|\alpha \right),\;\forall j\neq k. \end{array}$$ (26)

Computing protected exceedance probabilities, as defined in [19], also requires to run the HBI under the (prior) null hypothesis, *H*_0_, that there is no difference between models (i.e. *α*_0_ → ∞). The alternative hypothesis, *H*_1_, is the original case, in which *α*_0_ = 1. If we define *L* and *L*_0_ as the log-likelihood (actually the variational lower bound as its approximation) of all data given the model-space under *H*_1_ and *H*_0_, respectively, then the protected exceedance probability of *k*th model, ${\tilde{\phi }}_{k}$, is defined as:

$$\begin{array}{c} {\tilde{\phi }}_{k}=\phi_{k}\left(1-P_{0}\right)+\frac{1}{K}P_{0}, \end{array}$$ (27)

where

$$\begin{array}{c} P_{0}=\frac{1}{1+\exp(L-L_{0})}. \end{array}$$ (28)

Note that if *P*_0_ is close to 1, then model frequencies should be ignored, as the difference between models in the model space is due to chance. Furthermore, if data does not support any model, i.e. *P*_0_ is close to 1, then parameters should be estimated by fitting each model separately using the HBI.

## Supporting information

S1 Text Supplementary methods. (PDF)

S1 Fig A control analysis assessing the effects of prior variance on NHI performance. In scenario 1, similar to the analysis presented in the main text (Fig 3), 10 and 30 subjects generated with the RL and dual-*α* RL models, respectively. Conversely, in scenario 2, the RL model is more likely (30 subjects) than the dual-*α* RL model (10 subjects). In A and C, protected exceedance probability (PXP) as a function of prior variance is plotted in scenario 1 and 2, respectively. In B and D, estimation error for the learning rate parameter of RL is plotted in scenarios 1 and 2, respectively. The simulations show in general that no single prior is flexible enough to capture the different scenarios. In particular, while narrowing the prior reduces the complexity penalty (and thus somewhat improves model selection in scenario 1, when the more complex model should be favored), it also worsens parameter estimation in both scenarios. This is because the learning rates for the two models are, generatively, different, and a narrow prior cannot support both at once. Here, the true value of the RL learning rate was 0.1, which was quite away from the prior mean (i.e. 0.5), making it difficult for a narrower variance to capture it. Finally, this poor parameter estimation for the RL model has negative consequences also for model selection in scenario 2 (where the RL model should be favored, but the evidence for it is hampered by poor fit to the learning rate with smaller prior variance). The parameters used in this simulation are the same as those used in the original simulation analyses (Figs 3 and 4). Median across 100 simulations is plotted. Errorbars indicate the first and third quantiles. The prior variance in all simulation analyses of the main text is 6.25.
(TIF)

S2 Fig A control simulation analysis extending that from Fig 3, with different settings of learning rates for simulating data. The same parameters as in Fig 3 were used for simulations here, with the only difference that the learning rate parameter for the RL model was different here. In particular, the true learning rate of the RL was in the middle of those for the dual-*α* RL (for RL: *α* = 0.6; for dual-*α* RL: *α*^+^ = 0.8, *α*^−^ = 0.4). The difference between parameter estimation performance of the HPE and HBI is not as pronounced as in Fig 3, which is expected theoretically.
(TIF)

S1 Appendix Formal derivations of the HBI algorithm. (PDF)
