# Supplementary material for: Hierarchical Bayesian inference for concurrent model fitting and comparison for group studies
Source: PLoS Comput Biol. 2019 Jun 18;15(6):e1007043. doi: 10.1371/journal.pcbi.1007043 (PMC6581260; doi:10.1371/journal.pcbi.1007043)
Supplement: S1 Text — (PDF) [file pcbi.1007043.s001.pdf]

Supplementary Methods

Hierarchical Bayesian inference for concurrent model fitting and comparison for group studies

Payam Piray[1,*], Amir Dezfouli[2], Tom Heskes[3], Michael J. Frank[4] and Nathaniel D. Daw[1]

1. Princeton Neuroscience Institute, Princeton University
2. University of New South Wales Sydney
3. Institute for Computing and Information Sciences, Radboud University
4. Department of Cognitive, Linguistics, and Psychological Sciences, Brown University

* Corresponding author: Princeton Neuroscience Institute, Princeton University, Princeton, NJ 08540, USA. Email: ppiray@princeton.edu.

Simulation analyses

Here, we explain three models that were used frequently in our simulation analyses. For simulation analyses presented in Figures 1-2, data have been generated by two different models, Kalman filter (KF) and a reinforcement learning (RL) model. These models were also used in other simulations. Both models estimate the value of each action a_t, $Q_t(a_t)$, and take an action among two choices on every trial according to a softmax rule with the parameter $\beta > 0$. Both models learn using a prediction error signal, δ_t, the difference between the seen and expected reward:

$$\delta_t = r_t - Q_t(a_t)$$

where r_t is the reward. The RL model then updates its action value according to the prediction error:

$$Q_{t+1}(a_t) = Q_t(a_t) + \alpha\delta_t$$

where $0 < \alpha < 1$ is the learning rate. The update rule of the KL model is slightly different as it contains a dynamic learning rate (or Kalman gain), K_t, depending on the current estimate of variance, $V_t(a_t)$:

$$Q_{t+1}(a_t) = Q_t(a_t) + K_t\delta_t$$

where

$$K_t = \frac{V_t(a_t)}{V_t(a_t) + \omega}$$

where $\omega > 0$ is the observation noise. The variance also gets updated on every trial:

$$V_{t+1}(a_t) = (1 - K_t)V_t(a_t)$$

Note that all methods assume that parameters are normally distributed. Therefore, we used the sigmoid function, $\alpha = \sigma(\bar{\alpha}) = (1 + e^{-\bar{\alpha}})^{-1}$, to transform the normally-distributed learning rate, $\bar{\alpha}$, to the unit range. We used the exponential function to transform normally-distributed parameters corresponding to ω and β. For simulation analyses presented in Figures 1-2, these values were used as the group mean: RL, we used $\alpha = 0.1,\ \beta = 2$; Kalman filter $\omega = 2,\ \beta = 2$. For generating synthetic datasets for simulations, the parameters of the group of subjects assigned to each model was drawn from a normal distribution (with the standard deviation of 0.5). Next, for generating each individual dataset, we first generated two Gaussian random-walk (in the range of [-1 1]) sequences of 100 trials (corresponding to two actions). The learning rule of the corresponding model with the generated individual parameters were then used to generate a sequence of reward time-series for each action (both action values initialized at zero). Those reward time-series were then binarized and used to generate choice data given the corresponding model according to the softmax rule with the individual softmax parameter. The number of trials for all simulation was T=100, unless it is specified otherwise. The same procedure was employed for generating individual synthetic datasets in all simulations.

The dual-α RL model is similar to the RL model, with the only difference that two different learning rates, α^+ and α^- (both in the unit range), have been used for updating action values depending on whether the prediction error is positive or negative. Again, the sigmoid function was used to transform the normally distributed parameters into the unit range. For simulation analyses presented in Figures 3-6, these values were used as the group mean: RL, $\alpha = 0.1, \beta = 1$; dual-α RL: $\alpha^+ = 0.8, \alpha^- = 0.4, \beta = 3$.

For simulation analysis presented in Figure 7, these parameters used for the RL: $\alpha = 0.3, \beta = 3$. In scenario 1, the outliers were generated using the RL model with a very low decision noise parameter, i.e. $\alpha = 0.3, \beta = 0.1$. In scenario 2, the outliers were generated using RL with a low learning rate and a low decision noise parameter $\alpha = 0.1, \beta = 0.1$.

These parameters were used for all scenarios presented in Figure 8: the same parameters for the RL and the dual-α RL that used for generating data in Figures 3-6, the same parameters for the Kalman filter that used for generating data in Figure 1. These parameters used for the actor-critic RL: $\alpha^c = 0.1, \alpha^a = 0.6, \beta = 3$. For simulation analysis of the two-step task (Figure 9), we used parameters of the hybrid model reported in [1] (or the corresponding subset for the model-based or the model-free accounts) to generate data (201 trials). 30, 10 and 10 artificial subjects were generated using the hybrid, model-based and pure

model-free accounts. For generating the individual datasets, the outcome associated with each of the four second-stage options were generated according to a Gaussian random walk (the same sequences used in [2] were used here).

For the analysis presented in Figures 10A, 11A and 12A, data were generated using the same learning rate and decision parameters used for generating data in Figure 1. For the analysis presented in Figure 11B, data have been generated using the same parameters used for generating data in Figure 3 and the bias $b = 0$. In all simulations presented in Figures 10-11, the bias parameter, b, was generated using random draws form a normal distribution with the standard deviation 1. For the analysis presented in Figure 12, random numbers from the skewed distribution was drawn from the Pearson system of distributions with mean 0, variance 1, skewness –0.5 and kurtosis 3 (i.e. the kurtosis of the normal distribution) using the MATLAB function pearsrnd (Statistics and Machine Learning Toolbox).

Implementation of NHI and HPE

The NHI method is based on a Laplace approximation of the joint distribution of data and parameters, separately for each model and subject. Specifically, NHI relies on a maximum-a-posteriori (MAP) estimate of parameters. Formally, if μ_0 and V_0 are prior mean and variance, respectively, and $\ell(h) = p(x_n|M_k, h)N(h|\mu_0, V_0)$, in which x_n is the nth dataset, M_k denotes the kth model and h is the corresponding parameter vector (with the size D_k), then NHI quantifies model evidence of model k for subject n, L_{NHI}^{kn}, as:

$$L_{NHI}^{kn} = \log f_{kn} + \frac{1}{2} D_k \log 2\pi - \frac{1}{2} \log |A_{kn}|,$$

where,

$$\theta_{kn} = \mathrm{argmax_h}\, \ell(\mathrm{h})$$

$$A_{kn} = -\nabla\nabla \log\, \ell(\mathrm{h})\, |\theta_{kn}$$

$$f_{kn} = \ell(\theta_{kn}).$$

We then used values of L_{NHI}^{kn} across subjects to do the random-effects model selection and obtain model frequency given data and exceedance probability [3,4].

Similar to HBI and NHI, we used the Laplace approximation for quantifying model evidence by the HPE. Suppose that μ_k and V_k are group mean and variance, respectively in the last iteration of the algorithm (see [5,6] for a full explanation of the algorithm). We defined $\ell(h) = p(x_n|M_k, h)N(h|\mu_k, V_k)$

and obtained values of θ_{kn}, A_{kn} and f_{kn} using the same equations as above. Similar to [5,6], we used the Bayesian Information Criterion (BIC) to penalize models due to group parameters. Thus, the model evidence for the kth model, L_{HPE}^{k}, is given by the sum of (Laplace-approximated) log-evidence across all subjects plus the BIC-penalty of $2D_k$ (the mean and the diagonal variance) group parameters:

$$L_{HPE}^{k} = -D_k \log N + \sum_{n=1}^{N} \log f_{kn} + \frac{1}{2} D_k \log 2\pi - \frac{1}{2} \log |A_{kn}|,$$

Thus, the NHI relies on prior parameters μ_0 and V_0, which, for all models and parameters, were assumed μ_0=0 and $V_0 = 6.25$. This means that the prior mean for parameters constrained in the unit range, such as learning rate, was in the middle of their theoretical range. The value of prior variance is chosen to ensure that parameters can vary in a wide range with no substantial limitation due to the prior. In particular, a prior variance of 6.25 ensures that the difference between the maximum and minimum penalty is less than one chance-level choice in 90% of the theoretical range of a parameter constrained in the unit range. For such a parameter, the minimum penalty occurs at the mean, $\log N(0|0, V_0)$, and the penalty for $x = \text{logit}\,(0.95)$ is $\log N(x|0, V_0)$, where the logit function is the inverse of sigmoid function used for transforming unit-range parameters. Therefore, the relative penalty is given by

$$C(V_0) = \log N(x|0, V_0) - \log N(0|0, V_0)$$

Assuming that $C(V_0) = \log 0.5$, we obtain $V_0 = 6.25$. This value for prior variance also indicates that exponentially-transformed variables (such as decision noise) can vary between 0.05 and 19.00 with no substantial effect of prior (with the same criterion defined above).

These MAP estimates were also used to initialize both HPE and HBI methods. Non-linear derivative-based optimization method (Quasi-Newton algorithm as implemented in the fminunc routine in MATLAB, ©Mathwork, version R2014b) was used.

References

1. Daw ND, Gershman SJ, Seymour B, Dayan P, Dolan RJ. Model-based influences on humans' choices and striatal prediction errors. Neuron. 2011;69: 1204–1215. doi:10.1016/j.neuron.2011.02.027
2. Piray P, Toni I, Cools R. Human Choice Strategy Varies with Anatomical Projections from Ventromedial Prefrontal Cortex to Medial Striatum. J Neurosci. 2016;36: 2857–2867. doi:10.1523/JNEUROSCI.2033-15.2016
3. Stephan KE, Penny WD, Daunizeau J, Moran RJ, Friston KJ. Bayesian model selection for group studies. NeuroImage. 2009;46: 1004–1017. doi:10.1016/j.neuroimage.2009.03.025
4. Rigoux L, Stephan KE, Friston KJ, Daunizeau J. Bayesian model selection for group studies - revisited. NeuroImage. 2014;84: 971–985. doi:10.1016/j.neuroimage.2013.08.065

5. Huys QJM, Eshel N, O'Nions E, Sheridan L, Dayan P, Roiser JP. Bonsai trees in your head: how the pavlovian system sculpts goal-directed choices by pruning decision trees. PLoS Comput Biol. 2012;8: e1002410. doi:10.1371/journal.pcbi.1002410
6. Huys QJM, Cools R, Gölzer M, Friedel E, Heinz A, Dolan RJ, et al. Disentangling the roles of approach, activation and valence in instrumental and pavlovian responding. PLoS Comput Biol. 2011;7: e1002028. doi:10.1371/journal.pcbi.1002028
