# Supplementary figures and images for: Hierarchical Bayesian inference for concurrent model fitting and comparison for group studies
Source: PLoS Comput Biol. 2019 Jun 18;15(6):e1007043. doi: 10.1371/journal.pcbi.1007043 (PMC6581260; doi:10.1371/journal.pcbi.1007043)

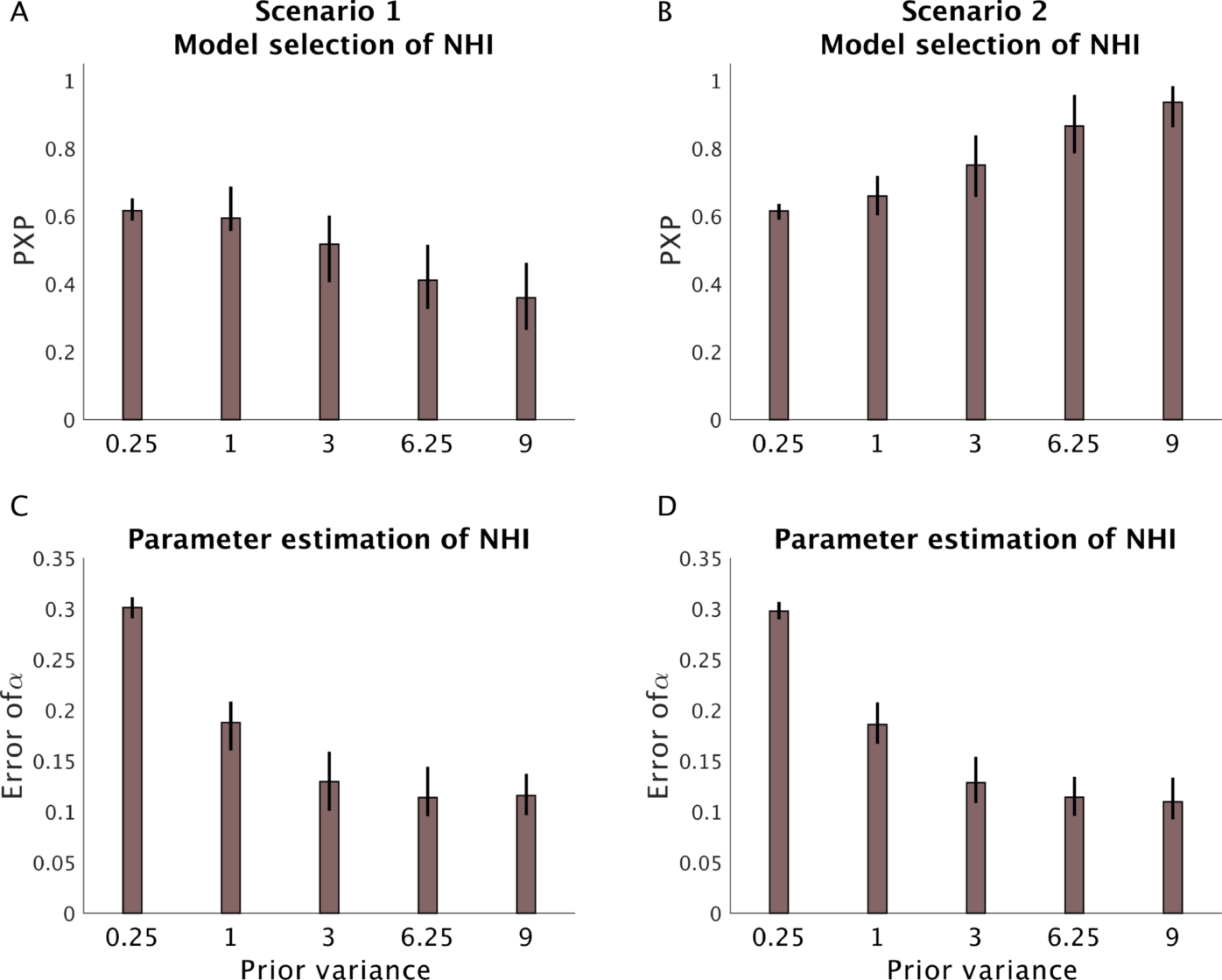

Supplement: S1 Fig — In scenario 1, similar to the analysis presented in the main text (Fig 3), 10 and 30 subjects generated with the RL and dual-α RL models, respectively. Conversely, in scenario 2, the RL model is more likely (30 subjects) than the dual-α RL model (10 subjects). In A and C, protected exceedance probability (PXP) as a function of prior variance is plotted in scenario 1 and 2, respectively. In B and D, estimation error for the learning rate parameter of RL is plotted in scenarios 1 and 2, respectively. The simulations show in general that no single prior is flexible enough to capture the different scenarios. In particular, while narrowing the prior reduces the complexity penalty (and thus somewhat improves model selection in scenario 1, when the more complex model should be favored), it also worsens parameter estimation in both scenarios. This is because the learning rates for the two models are, generatively, different, and a narrow prior cannot support both at once. Here, the true value of the RL learning rate was 0.1, which was quite away from the prior mean (i.e. 0.5), making it difficult for a narrower variance to capture it. Finally, this poor parameter estimation for the RL model has negative consequences also for model selection in scenario 2 (where the RL model should be favored, but the evidence for it is hampered by poor fit to the learning rate with smaller prior variance). The parameters used in this simulation are the same as those used in the original simulation analyses (Figs 3 and 4). Median across 100 simulations is plotted. Errorbars indicate the first and third quantiles. The prior variance in all simulation analyses of the main text is 6.25. (TIF) [file pcbi.1007043.s002.tif]

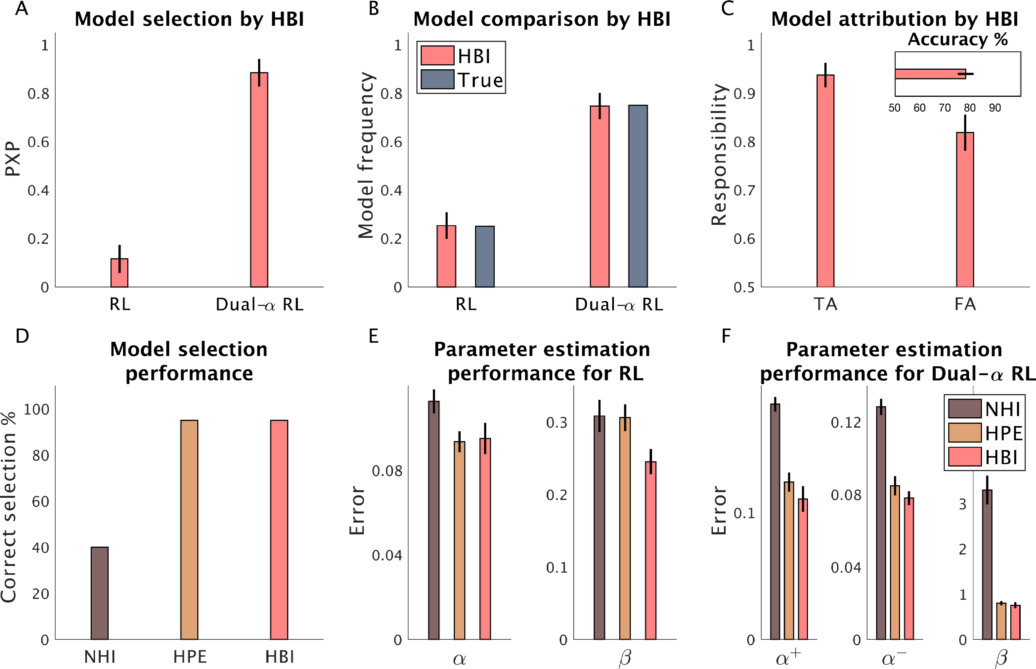

Supplement: S2 Fig — The same parameters as in Fig 3 were used for simulations here, with the only difference that the learning rate parameter for the RL model was different here. In particular, the true learning rate of the RL was in the middle of those for the dual-α RL (for RL: α = 0.6; for dual-α RL: α+ = 0.8, α− = 0.4). The difference between parameter estimation performance of the HPE and HBI is not as pronounced as in Fig 3, which is expected theoretically. (TIF) [file pcbi.1007043.s003.tif]
