# Supplementary material for: Hierarchical Bayesian inference for concurrent model fitting and comparison for group studies
Source: PLoS Comput Biol. 2019 Jun 18;15(6):e1007043. doi: 10.1371/journal.pcbi.1007043 (PMC6581260; doi:10.1371/journal.pcbi.1007043)
Supplement: S1 Appendix — (PDF) [file pcbi.1007043.s004.pdf]

Supplementary Appendix

Hierarchical Bayesian inference for model fitting and comparison

Payam Piray, Amir Dezfouli,
Tom Heskes, Michael J. Frank, Nathaniel D. Daw

1 Formal derivations of the HBI algorithm

In this appendix, we provide the proof of the results given in the main text. The proof is given in three parts by obtaining 1) the functional form of $q(\mathbf{H}, \mathbf{Z})$; 2) the posterior $q(\boldsymbol{\mu}, \boldsymbol{\tau}, \mathbf{m})$ and corresponding update equations; and 3) the update equations for the posterior over latent variables, $q(\mathbf{H}, \mathbf{Z})$.

Recall that the HBI generative model is given by

$$p(\mathbf{Z}|\mathbf{m}) = \prod_n \prod_k m_k^{z_{kn}}. \tag{1}$$

$$p(\mathbf{X}|\mathbf{H}, \mathbf{Z}) = \prod_k \prod_n p(\mathbf{x}_n|\mathbf{h}_{kn}, M_k)^{z_{kn}}, \tag{2}$$

$$p(\mathbf{H}|\mathbf{Z}, \boldsymbol{\mu}, \mathbf{T}) = \prod_k \prod_n \mathcal{N}(\mathbf{h}_{kn}|\boldsymbol{\mu}_k, \mathbf{T}_k^{-1})^{z_{kn}}, \tag{3}$$

$$p(\mathbf{m}) = \mathrm{Dir}(\mathbf{m}|\boldsymbol{\alpha_0}) = C(\alpha_0) \prod_{k=1}^{K} m_k^{\alpha_0 - 1}, \tag{4}$$

where $\mathbf{T}_k$ is a diagonal matrix. By defining $\mathbf{T}_k = \mathrm{diag}(\boldsymbol{\tau}_k)$, in which diag(.) is an operator outputting a diagonal matrix with elements given by $\boldsymbol{\tau}_k$, we have:

$$p(\boldsymbol{\mu}, \boldsymbol{\tau}) = \prod_{k=1}^{K} \mathcal{N}(\boldsymbol{\mu}_k|\mathbf{a}_0, \mathrm{diag}(b\boldsymbol{\tau}_k)^{-1})\mathcal{G}(\boldsymbol{\tau}_k|v, \mathbf{s}), \tag{5}$$

where we have defined:

$$\mathcal{G}(\boldsymbol{\tau}_k|v, \mathbf{s}) = \prod_{i=1}^{D_k} \mathcal{G}(\tau_{ki}|v, s).$$

Thus, the full probabilistic model is given by,

$$p(\mathbf{X}, \mathbf{H}, \mathbf{Z}, \boldsymbol{\mu}, \boldsymbol{\tau}, \mathbf{m}) = p(\mathbf{X}|\mathbf{H}, \mathbf{Z})p(\mathbf{H}|\mathbf{Z}, \boldsymbol{\mu}, \boldsymbol{\tau})p(\mathbf{Z}|\mathbf{m})p(\boldsymbol{\mu}|\boldsymbol{\tau})p(\boldsymbol{\tau})p(\mathbf{m}). \tag{6}$$

1.1 The functional form of the posterior over H and Z

Let us first consider the derivation of the functional form for the factor $q(\mathbf{H}, \mathbf{Z})$. According to standard results in variational inference [15,16], the log of this factor is given by:

$$\log q(\mathbf{H}, \mathbf{Z}) = \mathbb{E}_{\boldsymbol{\mu}, \boldsymbol{\tau}, \mathbf{m}}[\log p(\mathbf{X}, \mathbf{H}, \mathbf{Z}, \boldsymbol{\mu}, \boldsymbol{\tau}, \mathbf{m})] + \text{constant}^{\setminus \mathbf{H}, \setminus \mathbf{Z}},$$

where the constant term denotes all the terms independent of the corresponding variables. Note that the expectation is taken with respect to the current estimates of $q(\boldsymbol{\mu}, \boldsymbol{\tau}, \mathbf{m})$. By using equation (6) and absorbing all the terms which are independent of $\mathbf{H}$ and $\mathbf{Z}$ into the additive constant, we have:

$$\log q(\mathbf{H}, \mathbf{Z}) = \mathbb{E}_{\boldsymbol{\mu}, \boldsymbol{\tau}}[\log p(\mathbf{X}, \mathbf{H}|\mathbf{Z}, \boldsymbol{\mu}, \boldsymbol{\tau})] + \mathbb{E}_{\mathbf{m}}[\log p(\mathbf{Z}|\mathbf{m})] + \text{constant}^{\setminus \mathbf{H}, \setminus \mathbf{Z}}.$$

Substituting the two conditional distribution on the right-hand side using equations (1-3), we have:

$$\log q(\mathbf{H}, \mathbf{Z}) = \sum_k \sum_n z_{kn}(\log I_{kn} + \mathbb{E}_{\mathbf{m}}[\log m_k]) + \text{constant}^{\setminus \mathbf{H}, \setminus \mathbf{Z}},$$

where,

$$\log I_{kn} = \mathbb{E}_{\boldsymbol{\mu}, \boldsymbol{\tau}}[\log p(\mathbf{x}_n|\mathbf{h}_{kn}, M_k)\mathcal{N}(\mathbf{h}_{kn}|\boldsymbol{\mu}_k, \mathbf{T}_k^{-1})].$$

Note that we have defined $\mathbf{T}_k = \text{diag}(\boldsymbol{\tau}_k)$. We assume that there is a quadratic approximation of I_{kn} with respect to $\mathbf{h}_{kn}$,

$$I_{kn} \propto \exp(-\frac{1}{2}(\mathbf{h}_{kn} - \boldsymbol{\theta}_{kn})^{\mathsf{T}} \mathbf{A}_{kn}(\mathbf{h}_{kn} - \boldsymbol{\theta}_{kn})),$$

which gives,

$$\log q(\mathbf{H}, \mathbf{Z}) = \sum_k \sum_n z_{kn}(-\frac{1}{2}(\mathbf{h}_{kn} - \boldsymbol{\theta}_{kn})^\mathsf{T} \mathbf{A}_{kn}(\mathbf{h}_{kn} - \boldsymbol{\theta}_{kn}) + \mathbb{E}_{\mathbf{m}}[\log m_k])$$
$$+\text{constant}^{\backslash \mathbf{H}, \backslash \mathbf{Z}}.$$

Since $\log q(\mathbf{H}|\mathbf{Z}) = \log q(\mathbf{H}, \mathbf{Z}) - \log q(\mathbf{Z})$, we can read off terms involving $\mathbf{H}$ in $\log q(\mathbf{H}, \mathbf{Z})$ to obtain $\log q(\mathbf{H}|\mathbf{Z})$:

$$\log q(\mathbf{H}|\mathbf{Z}) = \sum_k \sum_n z_{kn}(-\frac{1}{2}(\mathbf{h}_{kn} - \boldsymbol{\theta}_{kn})^\mathsf{T} \mathbf{A}_{kn}(\mathbf{h}_{kn} - \boldsymbol{\theta}_{kn})) + \text{constant}^{\backslash \mathbf{H}}.$$

Requiring that this distribution should be normalized, we obtain:

$$q(\mathbf{H}|\mathbf{Z}) = \prod_k \prod_n \mathcal{N}(\mathbf{h}_{kn}|\boldsymbol{\theta}_{kn}, \mathbf{A}_{kn}^{-1})^{z_{kn}}. \tag{7}$$

Subtracting $\log q(\mathbf{H}|\mathbf{Z})$ from $\log q(\mathbf{H}, \mathbf{Z})$ cancels out the quadratic component and yields $\log q(\mathbf{Z})$, which is a linear function with respect to z_{kn}. Therefore, we have:

$$q(\mathbf{Z}) = \prod_k \prod_n r_{kn}^{z_{kn}}. \tag{8}$$

The functional form of $q(\mathbf{H}, \mathbf{Z})$ is then given by,

$$q(\mathbf{H}, \mathbf{Z}) = \prod_k \prod_n r_{kn}^{z_{kn}} \mathcal{N}(\mathbf{h}_{kn}|\boldsymbol{\theta}_{kn}, \mathbf{A}_{kn}^{-1})^{z_{kn}}. \tag{9}$$

Here our goal was to obtain the functional form of the posterior over latent variables. We will obtain values of r_{kn}, $\boldsymbol{\theta}_{kn}$ and $\mathbf{A}_{kn}$ in section 1.3.

1.2 The posterior over $\boldsymbol{\mu}$, $\boldsymbol{\tau}$ and $\mathbf{m}$

We continue with obtaining the functional form and update equations for the other variational factor $q(\boldsymbol{\mu}, \boldsymbol{\tau}, \mathbf{m})$. The posterior of $\mathbf{m}$ is independent from the posterior over $\boldsymbol{\mu}$ and $\boldsymbol{\tau}$ because the log-posterior decomposes into the terms that

only depend on $\mathbf{m}$ and terms that only depend on $\boldsymbol{\mu}$ and $\boldsymbol{\tau}$:

$$\begin{aligned}\log q(\boldsymbol{\mu}, \boldsymbol{\tau}, \mathbf{m}) &= \mathbb{E}_{\mathbf{H},\mathbf{Z}}[\log p(\mathbf{X}, \mathbf{H}, \mathbf{Z}, \boldsymbol{\mu}, \boldsymbol{\tau}, \mathbf{m})] + \text{constant}^{\setminus \boldsymbol{\mu},\boldsymbol{\tau},\mathbf{m}} \\ &= \mathbb{E}_{\mathbf{H},\mathbf{Z}}[\log p(\mathbf{H}|\mathbf{Z}, \boldsymbol{\mu}, \boldsymbol{\tau}) + \log p(\boldsymbol{\mu}, \boldsymbol{\tau})] + \mathbb{E}_{\mathbf{Z}}[\log p(\mathbf{Z}|\mathbf{m}) + p(\mathbf{m})] \\ &+ \text{constant}^{\setminus \boldsymbol{\mu},\boldsymbol{\tau},\mathbf{m}},\end{aligned}$$

where we have used equation (6). This implies that the variational posterior $q(\boldsymbol{\mu}, \mathbf{T}, \mathbf{m})$ factorizes to give $q(\boldsymbol{\mu}, \mathbf{T})q(\mathbf{m})$. Thus, the posterior over $\boldsymbol{\mu}$ and $\boldsymbol{\tau}$ is given by:

$$\log q(\boldsymbol{\mu}, \boldsymbol{\tau}) = \mathbb{E}_{\mathbf{H},\mathbf{Z}}[\log p(\mathbf{H}|\mathbf{Z}, \boldsymbol{\mu}, \boldsymbol{\tau})] + \log p(\boldsymbol{\mu}, \boldsymbol{\tau}) + \text{constant}^{\setminus \boldsymbol{\mu},\boldsymbol{\tau}},$$

in which we absorbed any terms independent of $\boldsymbol{\mu}$, $\boldsymbol{\tau}$ into the additive constant. Substituting for the distributions on the right-hand side, we have:

$$\begin{aligned}\log q(\boldsymbol{\mu}) =& \sum_k \sum_n \mathbb{E}_{\mathbf{H},\mathbf{Z},\boldsymbol{\tau}}[z_{kn} \log \mathcal{N}(\mathbf{h}_{kn}|\boldsymbol{\mu}_k, \mathbf{T}_k^{-1})] + \\ & \sum_k \log \mathcal{N}(\boldsymbol{\mu}_k|\mathbf{a}_0, (b\mathbf{T}_k)^{-1}) + \sum_k \log \mathcal{G}(\boldsymbol{\tau}_k|v, \mathbf{s}) + \text{constant}^{\setminus \boldsymbol{\mu},\boldsymbol{\tau}}.\end{aligned}$$

Note that we have defined $\mathbf{T}_k = \text{diag}(\boldsymbol{\tau}_k)$. Using equation (9) and by absorbing terms independent of $\boldsymbol{\mu}_k$ and $\boldsymbol{\tau}_k$ into the additive constant, we have:

$$\begin{aligned}\log q(\boldsymbol{\mu}_k, \boldsymbol{\tau}_k) =& \sum_n \frac{1}{2} r_{kn} \log |\mathbf{T}_k| - \sum_n \frac{1}{2} (\boldsymbol{\mu}_k - \boldsymbol{\theta}_{kn})^{\mathsf{T}} r_{kn} \mathbf{T}_k (\boldsymbol{\mu}_k - \boldsymbol{\theta}_{kn}) + \\ & - \sum_n \frac{1}{2} r_{kn} \text{Tr}(\mathbf{A}_{\text{kn}}^{-1} \mathbf{T}_{\text{k}}) + \frac{1}{2} \log |\mathbf{T}_k| + \\ & - \frac{1}{2} (\boldsymbol{\mu}_k - \mathbf{a}_0)^{\mathsf{T}} b \mathbf{T}_k (\boldsymbol{\mu}_k - \mathbf{a}_0) + \\ & + \sum_{i=1}^{D_k} \log G(v, s) + (v - 1) \log \tau_{ki} - s\tau_{ki} + \text{constant}^{\setminus \boldsymbol{\mu}_k,\boldsymbol{\tau}_k}.\end{aligned}$$

As the right-hand side is quadratic with respect to $\boldsymbol{\mu}_k$, the posterior over $\boldsymbol{\mu}_k$ also takes the form of a Gaussian with a variance depending on $\boldsymbol{\tau}_k$:

$$q(\boldsymbol{\mu}_k|\boldsymbol{\tau}_k) = \mathcal{N}(\boldsymbol{\mu}_k|\mathbf{a}_k, (\beta_k \mathbf{T}_k)^{-1}),$$

where

$$\mathbf{a}_k = \frac{1}{\overline{N}_k + b} (\sum_n r_{kn} \boldsymbol{\theta}_{kn} + b\mathbf{a}_0)$$

$$\beta_k = b + \overline{N}_k,$$

and N_k is given by:

$$\overline{N}_k = \sum_n r_{kn}.$$

By subtracting $\log q(\boldsymbol{\mu}_k|\boldsymbol{\tau}_k)$ from $\log q(\boldsymbol{\mu}_k, \boldsymbol{\tau}_k)$, we obtain the posterior over $\boldsymbol{\tau}_k$:

$$q(\boldsymbol{\tau}_k) = \mathcal{G}(\boldsymbol{\tau}_k|\nu_k, \boldsymbol{\sigma}_k),$$

where

$$\begin{aligned}\boldsymbol{\sigma}_k =& \frac{1}{2}\sum_n \mathrm{diag}(r_{kn}[(\boldsymbol{\theta}_{kn} - \overline{\boldsymbol{\theta}}_k)(\boldsymbol{\theta}_{kn} - \overline{\boldsymbol{\theta}}_k)^\mathsf{T} + \mathbf{A}_{kn}^{-1}]) \\ &+ \frac{1}{2}\frac{b\overline{N}_k}{b + \overline{N}_k}\mathrm{diag}((\overline{\boldsymbol{\theta}}_k - \mathbf{a}_0)(\overline{\boldsymbol{\theta}}_k - \mathbf{a}_0)^\mathsf{T}) + \mathbf{s}\end{aligned}$$

$$\nu_k = v + \frac{1}{2}\overline{N}_k,$$

and $\overline{\boldsymbol{\theta}}_k$ is given by:

$$\overline{\boldsymbol{\theta}}_k = \frac{1}{\overline{N}_k}\sum_n r_{kn}\boldsymbol{\theta}_{kn}.$$

Finally, we consider the factor $q(\mathbf{m})$:

$$\log q(\mathbf{m}) = \mathbb{E}_{\mathbf{Z}}[\log p(\mathbf{Z}|\mathbf{m})] + \log p(\mathbf{m}) + \mathrm{constant}^{\backslash \mathbf{m}}.$$

Substituting for the two distributions on the right-hand side, we have

$$\log q(\mathbf{m}) = \sum_k \sum_n r_{kn} \log m_k + \log C(\alpha_0) + \sum_k (\alpha_0 - 1)\log m_k + \mathrm{constant}^{\backslash \mathbf{m}}.$$

Therefore $q(\mathbf{m})$ takes the form of Dirichlet distribution:

$$q(\mathbf{m}) = \mathrm{Dir}(\mathbf{m}|\boldsymbol{\alpha}),$$

where $\boldsymbol{\alpha}$ has components α_k given by,

$$\alpha_k = \alpha_0 + \overline{N}_k.$$

1.3 The posterior over H and Z

We have already seen in section 1.1 that $q(\mathbf{H}, \mathbf{Z})$ could be written,

$$\log q(\mathbf{H}, \mathbf{Z}) = \sum_k \sum_n z_{kn}(\log I_{kn} + \mathbb{E}_{\mathbf{m}}[\log m_k]) + \text{constant}^{\backslash \mathbf{H}, \backslash \mathbf{Z}},$$

where,

$$\log I_{kn} = \mathbb{E}_{\boldsymbol{\mu},\boldsymbol{\tau}}[\log p(\mathbf{x}_n|\mathbf{h}_{kn}, M_k)\mathcal{N}(\mathbf{h}_{kn}|\boldsymbol{\mu}_k, \mathbf{T}_k^{-1})].$$

Since we have already obtained $q(\boldsymbol{\mu}, \boldsymbol{\tau})$, we can now compute I_{kn}:

$$\begin{aligned}\log I_{kn} = &+ \log p(\mathbf{x}_n|\mathbf{h}_{kn}, M_k) - \frac{1}{2}D_k \log 2\pi + \frac{1}{2}\mathbb{E}[\log |\mathbf{T}_k|] \\ &- \frac{1}{2}\mathbb{E}_{\mathbf{T},\boldsymbol{\mu}}[(\mathbf{h}_{kn} - \boldsymbol{\mu}_k)^\mathsf{T}\mathbf{T}_k(\mathbf{h}_{kn} - \boldsymbol{\mu}_k)].\end{aligned}$$

Therefore, $\log I_{kn}$ is given by:

$$\begin{aligned}\log I_{kn} = &\log p(\mathbf{x}_n|\mathbf{h}_{kn}, M_k) - \frac{1}{2}D_k \log 2\pi + \frac{1}{2}\mathbb{E}[\log |\mathbf{T}_k|] \\ &- \frac{1}{2}(\mathbf{h}_{kn} - \mathbf{a}_k)^\mathsf{T}\mathbb{E}[\mathbf{T}_k](\mathbf{h}_{kn} - \mathbf{a}_k) - \frac{1}{2}\mathbb{E}[\text{Tr}(\mathbf{T}_\text{k}(\beta_\text{k}\mathbf{T}_\text{k})^{-1})],\end{aligned}$$

which can be written in the form,

$$\log I_{kn} = \log p(\mathbf{x}_n|\mathbf{h}_{kn}, M_k)\mathcal{N}(\mathbf{h}_{kn}|\mathbf{a}_k, \mathbb{E}[\mathbf{T}_k]^{-1}) + \lambda_k,$$

where λ_k is independent of $\mathbf{h}_{kn}$ and is given by

$$\lambda_k = \frac{1}{2}\mathbb{E}[\log |\mathbf{T}_k|] - \frac{1}{2}\log |\mathbb{E}[\mathbf{T}_k]| - \frac{1}{2}\frac{D_k}{\beta_k},$$

Substituting the moments of $\mathbf{T}_k = \text{diag}(\boldsymbol{\tau}_k)$ with their values under $q(\boldsymbol{\tau}_k)$,

$$\log \mathbb{E}[\boldsymbol{\tau}_k] = D_k \log \nu_k - \sum_i \log \sigma_{ki},$$

$$\mathbb{E}[\log \boldsymbol{\tau}_k] = D_k \psi(\nu_k) - \sum_i \log \sigma_{ki},$$

gives

$$\lambda_k = \frac{D_k}{2}\big(\psi(\nu_k) - \log \nu_k - \frac{1}{\beta_k}\big)$$

Now, we make a quadratic approximation of $p(\mathbf{x}_n|\mathbf{h}_{kn}, M_k)\mathcal{N}(\mathbf{h}_{kn}|\mathbf{a}_k, \mathbb{E}[\mathbf{T}_k]^{-1})$ with respect to $\mathbf{h}_{kn}$ (for example using Laplace approximation or any other method):

$$p(\mathbf{x}_n|\mathbf{h}_{kn}, M_k)\mathcal{N}(\mathbf{h}_{kn}|\mathbf{a}_k, \mathbb{E}[\mathbf{T}_k]^{-1}) \simeq \\ f_{kn} \exp(-\frac{1}{2}(\mathbf{h}_{kn} - \boldsymbol{\theta}_{kn})^\mathsf{T}\mathbf{A}_{kn}(\mathbf{h}_{kn} - \boldsymbol{\theta}_{kn})).$$

Substituting this approximation into $\log I_{kn}$, we obtain

$$\log I_{kn} = \log f_{kn} - \frac{1}{2}(\mathbf{h}_{kn} - \boldsymbol{\theta}_{kn})^\mathsf{T}\mathbf{A}_{kn}(\mathbf{h}_{kn} - \boldsymbol{\theta}_{kn}) + \lambda_k.$$

Therefore, we have:

$$\log q(\mathbf{H}, \mathbf{Z}) = \sum_k \sum_n z_{kn}\Big(+ \log f_{kn} - \frac{1}{2}(\mathbf{h}_{kn} - \boldsymbol{\theta}_{kn})^\mathsf{T}\mathbf{A}_{kn}(\mathbf{h}_{kn} - \boldsymbol{\theta}_{kn}) \\ + \lambda_k + \mathbb{E}[\log m_k]\Big) + \text{constant}^{\backslash\mathbf{H},\backslash\mathbf{Z}}.$$

Subtracting this equation from $\log q(\mathbf{H}|Z)$ given by the log of equation (7), we have:

$$\log q(\mathbf{Z}) = \sum_k \sum_n z_{kn} \log \rho_{kn} + \text{constant}^{\backslash\mathbf{H},\backslash\mathbf{Z}},$$

where

$$\log \rho_{kn} = \log f_{kn} + \frac{1}{2}D_k \log 2\pi - \frac{1}{2}\log|\mathbf{A}_{kn}| + \lambda_k + \mathbb{E}[\log m_k].$$

Requiring that $q(\mathbf{Z})$ be normalized, we obtain equation (8), where

$$r_{kn} = \frac{\rho_{kn}}{\sum_{j=1}^K \rho_{jn}},$$

which completes the proof.
